# Novel zoonotic cases of *Plasmodium simium* from São Paulo with a reference genome of the Brazilian strain

**DOI:** 10.1038/s41598-025-27554-0

**Published:** 2025-12-12

**Authors:** Emilia Manko, Nina Billows, Matthew Higgins, Daniel Ward, Amy Ibrahim, Susana Campino, Silvia Maria Di Santi, Taane G. Clark

**Affiliations:** 1 Faculty of Infectious & Tropical Diseases, London School of Hygiene & Tropical Medicine, London, UK; 2 Infectious Diseases Department, University of São Paulo, São Paulo, 05403-000 Brazil; 3 Malaria Reference Laboratory, Instituto Adolfo Lutz, São Paulo, Brazil; 4 Faculty of Epidemiology & Population Health, London School of Hygiene & Tropical Medicine, London, UK

**Keywords:** Evolution, Infectious diseases, Malaria, Mitochondrial genome, Genome, Genetics, Genomics, Comparative genomics

## Abstract

Zoonotic malaria, caused by parasites like *Plasmodium simium*, poses challenges to control and elimination efforts due to undetected reservoirs and altered transmission dynamics. Recent outbreaks in Brazil underscore the importance of *P. simium* spillovers from non-human primates. Genomic studies are crucial for identifying molecular markers of host adaptation and improving diagnostic tools; however, a high-quality reference genome is needed. To address this, we used Oxford Nanopore and Illumina sequencing platforms to generate a high-quality *P. simium* reference (Ps_SãoPaulo, 21.8 Mb). Comparative genomic analyses confirmed known differences with *P. vivax*, including mitochondrial mutations, deletions in reticulocyte-invasion genes (*RBP2a, DBP*), and variants in blood-stage antigen genes (*msp3.1, msp3.2*). Genome-wide analyses of 38 *P. simium* samples from Brazil revealed expected divergence from *P. vivax* and potential unique signatures of selection and host adaptation. This study provides the most comprehensive *P. simium* reference genome, advancing our understanding of its genomic diversity, with insights for malaria control in Brazil.

**Supplementary Information:**

The online version contains supplementary material available at 10.1038/s41598-025-27554-0.

## Introduction

Malaria, caused by *Plasmodium* parasites, resulted in 263 million cases and 597,000 deaths in 2023^1^. The occurrence of *Plasmodium* parasites in zoonotic hosts, such as Non-Human Primates (NHPs) can lead to undetected reservoirs of malaria that can contribute to small outbreaks when transmitted to humans^2^. South America and Asia are the main hotspots where recent natural human infections by zoonotic *Plasmodium* parasites have been confirmed across several NHP malaria species. This includes *Plasmodium simium*, a malaria parasite identified in howler monkeys (*Alouatta guariba*) in southeastern Brazil. In regions where *Plasmodium vivax* is absent, zoonotic spillover of *P. simium* to humans has been documented, including a notable outbreak in São Paulo and Rio de Janeiro between 2015 and 2016, originating from an emerging hotspot in the Atlantic Forest^3–5^. Advancements in molecular techniques allow for better surveillance of zoonotic spillover infections in humans living or working closely with the natural habitat of NHP hosts^6^. However, there is a need for an increased geographical sampling of high-quality genomes of closely related human and simian malaria isolates, and a resulting comparative genomic analysis^7^.

Comparative genomic studies confirmed *P. simium* isolates are closely related to *P. vivax,* with an estimated time of divergence within the last 500 years^8^. The similarity between *P. vivax* and *P. simium* can lead to misclassification and misdiagnosis. Analyses confirmed that *P. simium* isolates infecting humans and monkeys possess identical mitochondrial sequences, distinguished from *P. vivax* by only two single nucleotide polymorphisms (SNPs) (PvP01_v1: MtDNA 4133 T- > C, 4467 A- > G)^9^. Studies also observed low genetic diversity in *P. simium* compared to *P. vivax*, and higher genetic diversity between *P. simium* parasites isolated from humans compared to those sourced from monkeys^8,10^. Taken together, these findings fit the hypothesis that *P. simium* infected NHPs because of a host switch of *P. vivax* from humans, and a recent American origin, probably within the last 500 years^10^. Whether single or multiple independent transfer events have occurred is unclear. However, analysis of genes involved in erythrocyte invasion has revealed deletions in *reticulocyte-binding protein 2a* (*RBP2a*) and *Duffy-binding protein 1 (DBP)* genes that could suggest genomic signatures of adaptation to new hosts^8,10^.

With the recent advancement of third-generation sequencing technologies, draft reference genomes for understudied simian isolates, like *P. brasilianum and P. knowlesi*^11–13^, have been released. Although some genome assemblies have been released, a high-quality reference genome for *P. simium* is still lacking. Mourier et al. made an important contribution by providing the first genome assembly for *P. simium* using short-read Illumina data, comprising 2191 scaffolds (> 1 kb)^8^. However, this assembly remains fragmented and does not offer the level of resolution attainable with long-read sequencing approaches. In our work, we extend the current genomic knowledge of *P. simium* by sequencing 12 genomes sourced from the Atlantic Forest region in São Paulo, Brazil, and create a higher-quality draft genome for *P. simium* using long-read Oxford Nanopore Technologies (ONT) and Illumina sequencing; thereby assisting future studies of *P. simium* evolution and genomic diversity, not dependent on *P. vivax* reference genomes.

## Results

### New *P. simium* sequence data and reference genome

Twelve *P. simium* isolates from the São Paulo region (2013–2020) underwent Illumina sequencing. The average coverage of *P. simium* isolates (mapped to PvP01) in the core nuclear genome was 11.0-fold (range: 5.9- to 23.7-fold) and in the mitochondria was 24.2-fold (range: 1.5- to 42.8-fold) (Table S1). Three isolates underwent ONT sequencing (mean read length of 7322 bp), yielding an average of 116,840 high-quality reads, with mean coverage in the core nuclear genome of 20.5-fold (min: 17.1-fold; max: 24.3-fold) and in the mitochondria of 34.1-fold (Table S1). Highly variable regions (e.g. sub telomeric regions) had low coverage (Short reads: 6.5-fold; Long reads: 6.1-fold) (Figure S1).

Using a robust pipeline (Figure S2), combined filtered short (6,936,751; 71.5% of all merged) and long (312,352; 89.11% of all merged) reads for three high-quality isolates were assembled using a hybrid approach. This generated 3932 contigs, which were polished and gap-filled before undergoing scaffolding. Scaffolding links and orders contigs into larger scaffolds using additional information, such as paired-end or long-range data, to establish their relative orientation. While scaffolds may still contain gaps where the sequence is unknown, they better approximate the chromosome structure. This process, guided by the *P. vivax* PvP01_v1 reference genome (28.9 Mb; 24.2 Mb excluding unplaced contigs), resulted in improved contiguity and overall assembly quality. Scaffolds shorter than 500 bp were discarded before predicting annotations. The final reference consists of 105 scaffolds: 14 nuclear chromosomes, one mitochondrial sequence and 90 supporting contigs (size: 21.8 Mb; 21.5 Mb excluding unplaced contigs) (Table 1). Since the new *P. simium* assembly was generated using a chimeric approach combining three samples, we evaluated each sample’s contribution by identifying genomic regions with elevated coverage (> 2.5-fold) unique to a single sample, as well as reference alleles observed exclusively in one sample. In total, 140 regions showed greater coverage from one sample (ERR10301330: 127 regions; ERR10301323: seven regions; ERR10301333: six regions), indicating that ERR10301330 contributed the most to the assembly despite the use of three samples (Table S2). This conclusion was further supported by identifying ‘single-sample’ reference alleles, with 1473 such alleles observed overall (ERR10301330: 670; ERR10301323: 351; ERR10301333: 452 unique reference alleles) (Figure S3).

**Table 1:** Summary of the complete de novo draft genomes compared to *P. vivax* PvP01***.***

Metric	*P. simium* Ps_SãoPaulo	P. vivax P01**
Size (Mb)	21.8	28.9
Size chromosomes (Mb)*	21.7	24.2
GC content (%)	45.1	39.8
Scaffolds and contigs	93	226
Genes (n)	4,849	6,642
N50 (bp)	1,939,510	1,758,561
Detected orthologues (%)	94.0	97.2

* does not include the apicoplast for Ps_SãoPaulo; ** Auburn et al. (2016).

### Comparison to the *P. vivax* genome and *P. simium* assembly

**Fig. 1:**
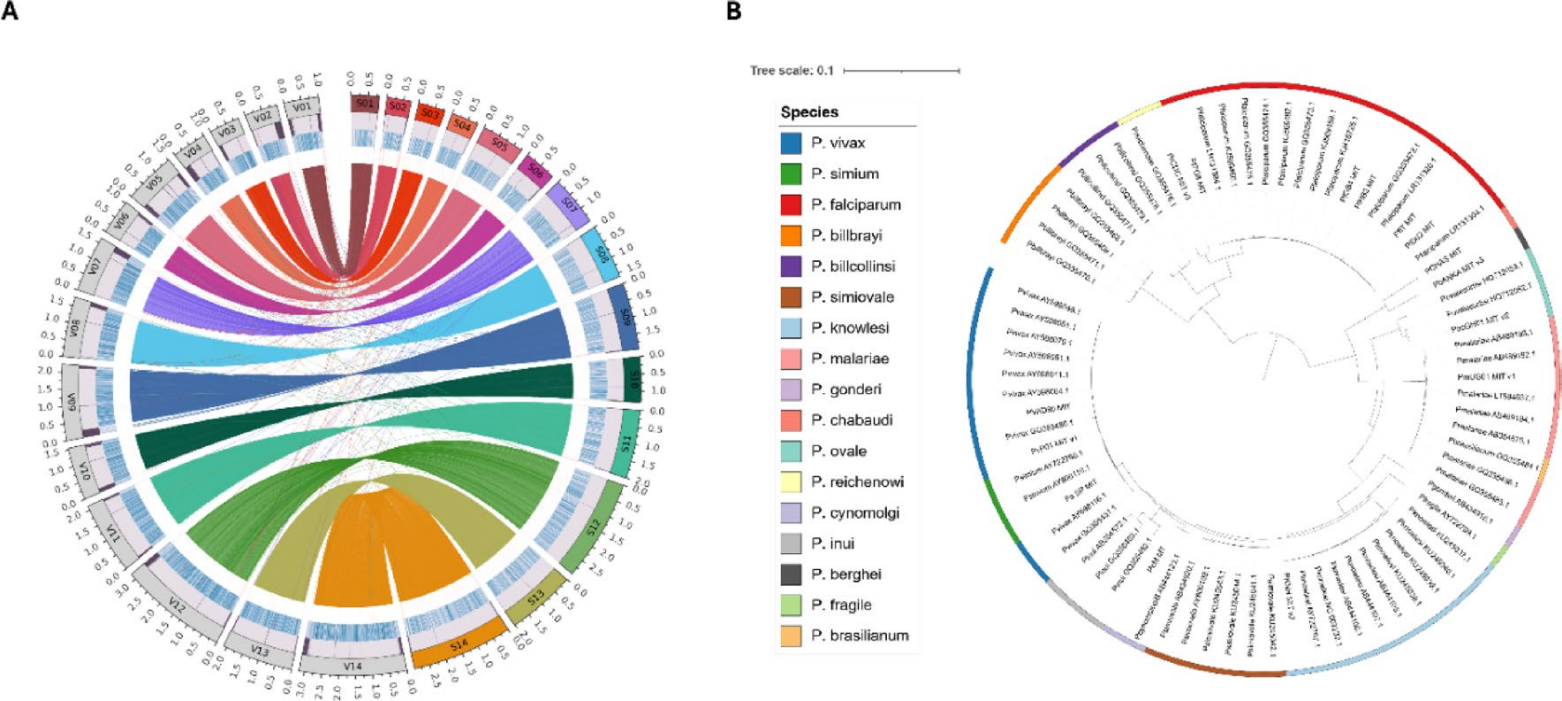
Genomic landscape of *P. simum* Ps_SãoPaulo (Ps SP) assembly. (**A**) Circos plot of *P. vivax* reference (left side; V01-V14) and *P. simium São Paulo* (Ps SP) (right side; S01-S14) assemblies with annotation. From the outer to the inner, the circular tracks represent pseudochromosomes with Mb scale, GC content (1–58 per 5 kb), gene density (0–5 per 5 kb), identified gaps, and synteny links. Each center line represents a pair of homologous sequences between two genomes. The size of 21.8 Mb of the genome is represented by a total of 3,247 homologous fragments of an average identity of 98.2%. Small translocations visible in the background of the circos plot; (**B**) A maximum-likelihood tree showing the relationship of human (N = 51) and non-human Plasmodium (N = 24) isolates in relation to *P. simium São Paulo* (Ps SP) (bold) using mitochondrial sequences. Clusters of species are as expected. Tree is rooted using the midpoint.

We first compared the new *P. simium “*Ps_SãoPaulo*”,* draft genome to an existing *P. simium* assembly that was previously created using short-read sequence data (GCA_913736665)^8^. The new draft genome has high synteny with the short-read *P. simium* assembly but has greater completeness (Figure S4). Furthermore, we also compared the *P. simium* draft genome to *P. vivax* PvP01. The chromosome sizes are on average 11% (standard deviation: 7%) shorter compared to *P. vivax* PvP01*,* due to a lack of coverage of sub-telomeric regions. *P. simium* chromosomes 1, 2, and 4 show notable differences in length and have high sub-telomeric content, with fractions of 23%, 32%, and 29%, respectively. The Ps_SãoPaulo draft genome has higher GC content (45.1%) compared to *P. vivax* PvP01 (39.6%), but after removing AT-rich sub-telomeric regions from PvP01, its GC content is similar (45.7%) (Figure S5). Pairwise whole genome alignment revealed conservation and differences between Ps_SãoPaulo and PvP01. On average, 98.1% of nucleotide positions were identical between the two aligned DNA sequences (sequence homology) across 14 chromosomes and 99.8% for the mitochondrion (Table S3). The synteny between *P. vivax* PvP01 and Ps_SãoPaulo scaffolds is high, with minor translocations and inversions present in the new assembly (Fig. 1A, Table S4). To assess the accuracy of these structural variants, we mapped long reads from all three contributing samples and evaluated support for each rearrangement. Specifically, we calculated the average read depth across the region, the average number of bases covered per read, and the average fraction of the region covered by reads (Table S4).

Regions with very low average coverage or minimal read support were considered poorly supported and potentially misassembled. For example, regions on Ps_SP_9 and the first region on Ps_SP_14 showed zero coverage across all three samples, while the inversion on Ps_SP_7 had minimal coverage (0.33-fold average depth; 3.7 bp average overlap; 3.2% covered). Therefore, three structural variants (Ps_SP_9, Ps_SP_14 [11145–11379], and Ps_SP_7) were classified as poorly supported and may represent mis-assemblies or unresolved rearrangements in the current scaffold set.

Furthermore, a comparison of the Ps_SãoPaulo mitochondrial genome (MtDNA; 5989 bp) to its PvP01 counterpart confirmed nucleotide differences at positions 4133 and 4467 in the mitochondrial genome (assembly PvP01) and revealed five previously unreported SNPs at positions 181, 2036, 2474, 4510, and 4640 bp. These SNPs are also present in the Peruvian Amazon (PvPAM) strain, confirming they are specific to South America and not exclusive to *P. simium*^14^. The Ps_SãoPaulo genome had 94.0% (N = 3424/3642) complete gene orthologues, with 3.3% being fragmented and 2.7% missing. The Companion software suite annotated 4910 genes, comprising 4852 protein-coding genes (including three mitochondrial genes) and 58 non-protein-coding RNA genes, such as tRNAs and rRNAs. In total, 5165 polypeptides were predicted (Figure S6). Each gene may encode multiple polypeptides due to alternative splicing, gene duplication or the presence of paralogous gene copies. Therefore, the total number of predicted polypeptides can exceed the number of unique “coding” genes, as one gene can give rise to several distinct polypeptide products. Orthologous mappings were extracted from *P. simium* polypeptides, and statistics were compared against protein-coding genes of *P. vivax* PvP01 (N = 6523). We identified 141 novel polypeptides not annotated with any *P. vivax* PvP01 orthologue protein-coding gene, although there were BLAST (nucleotide) hits with *P. vivax* PvP01 for fragments of sequences*.* Unresolved gaps or mis-assemblies in the scaffolds prevented the assignment of gene annotations in certain regions of the genome. Annotation of the remaining polypeptides (N = 5024) overlaps with 75.7% of the protein-coding *P. vivax* gene content. Unidentified protein-coding *P.* vivax genes (24.3%) were predominantly located in PvP01 contigs that are not considered as core genome.

### Comparative analysis across *Plasmodium* species

We also investigated differences of *P. simium* to other *Plasmodium* species. Firstly, a maximum-likelihood tree, based on an alignment of mitochondrial nucleotide sequences, confirmed the new Ps_SãoPaulo reference (MtDNA) clusters as expected with other *P. simium* mitochondria sequences and *P. vivax* (Fig. 1B)^11^. Separately, using a multiple sequence alignment of 4998 *P. vivax* PvP01 proteins and *P. simium* Ps_SãoPaulo orthologue polypeptides*,* we compared their conservation and homology across species. Proteins with any uncertain amino acid predictions were excluded from further analysis. The Jaccard similarity index was used to quantify the proportion of shared elements (e.g., genes or features) between datasets, providing a measure of overlap or similarity. A total of 62% (3067/4970) had equal sequence size and an average Jaccard similarity of 0.99 (standard deviation (SD): 0.01). The remaining proteins (N = 1903) varied in size, but the average Jaccard similarity for pairwise comparison was 0.91 (SD: 0.16), which reflects high similarity and conservation between the gene content of *P. simium* and *P. vivax.*

The comparative analysis was extended to *Plasmodium* genes of broad interest, including those related to vertebrate host and vector invasion, and drug resistance (N = 134). The majority (85%) of these genes had an orthologous *P. simium* protein that allowed for comparative analysis (Table S5). No large indels were found in genes linked to drug resistance. We observed small indels in the merozoite surface protein MSP3.1 and MSP3.2, as well as the circumsporozoite protein (CSP), which are candidates for malaria vaccines. A few indels were detected in the Tryptophan Rich Antigens (TRAgs) multi-gene family, specifically TRAG12 and TRAG13, as well as TSR3. Lastly, Merozoite Adhesive Erythrocytic Binding-Like Protein (MAEBL), responsible for invading red blood cells, and CTRP, which is responsible for ookinete motility and mosquito midgut invasion, had a few small deletions. As expected, deletions were identified in RBP2a (1003 amino acids) and DBP (9 and 91 amino acids) (Figure S7). The larger 91-amino acid deletion in DBP is found only in the new *P. simium* reference (Ps_SãoPaulo). Meanwhile, the shorter 9-amino acid deletion in DBP appears to be fixed in *P. vivax* Sal I and *P. cynomolgi*, occurring between residues 684A and 694E (Figure S8).

### New P. simium isolates in the context of South American P. vivax

**Fig. 2:**
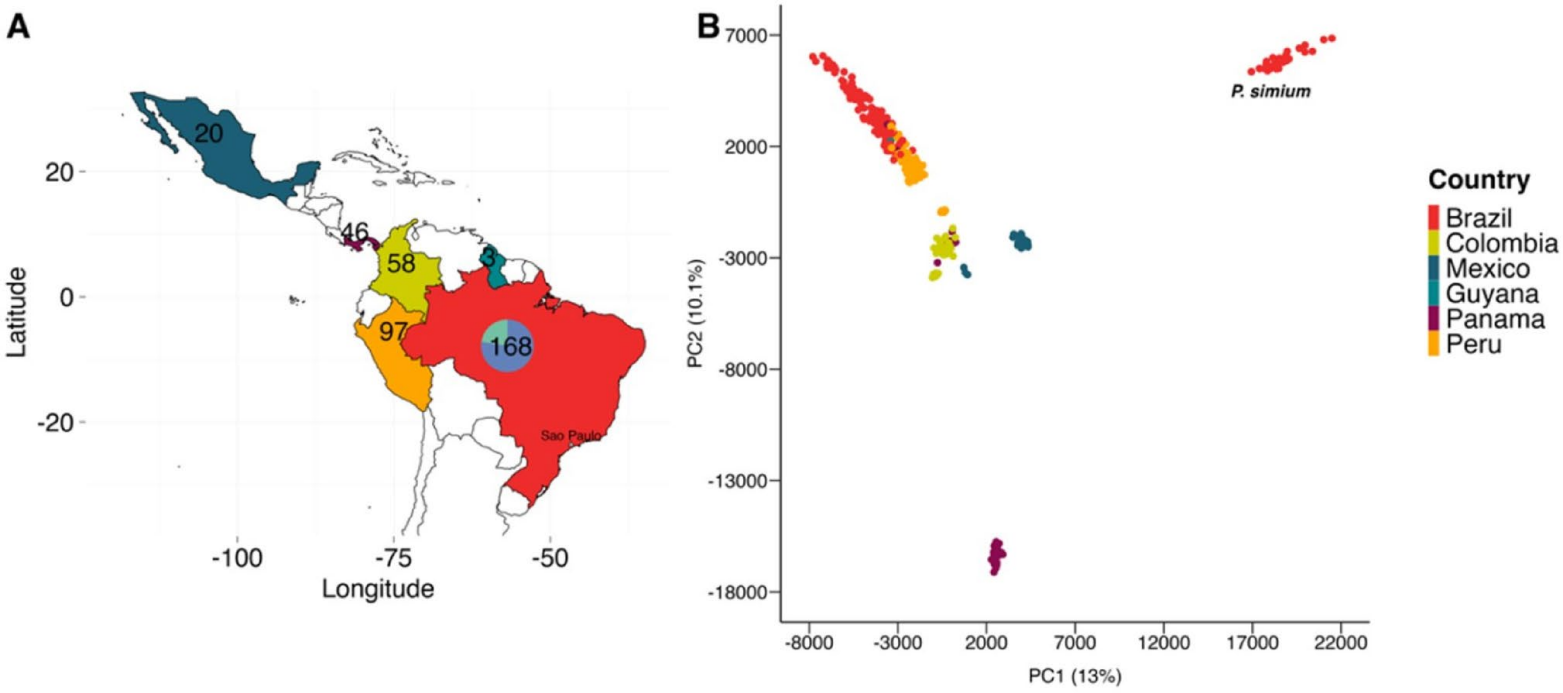
Population structure of 392 *P. simium* and *P. vivax* isolates across South America. (**A**) Map with the color coordinated geographic locations and populations sizes (*P. vivax*: Mexico = 20, Colombia = 58, Peru = 97; Panama = 46, Guyana = 3, Brazil = 130; *P. simium:* Brazil = 38) (**B**) Multidimensional scaling of first two components with annotated cluster of *P. simium* isolates.

To gain insight into the geographical context of *P. simium* in South America, we performed a population structure analysis of 38 isolates (12 new, 26 public^8,10^) with 354 high-quality *P. vivax* isolates (Table S6; Fig. 2A)^15,16^. The analysis used 118,573 bi-allelic chromosomal SNPs and revealed that Brazilian *P. simium* isolates form a unique cluster (Fig. 2B). A comparison of *P. simium* and *P. vivax* mitochondrial sequences confirmed the known *P. simium-*specific haplotype with a cytosine at 4133 and guanine at 4467 positions (relative to *P. vivax* PvP01), in all isolates with sufficient coverage (> tenfold) (Figure S9). A further mutation at position 2474 was observed only in six new *P. simium* isolates, as well as in *P. vivax* isolates across South America (Table S7).

Furthermore, we compared the nucleotide diversity of *P. simium* to *P. vivax* from Brazil. *P. simium* samples had overall low nucleotide diversity (π) when compared to *P. vivax* isolates from Brazilian districts (Wilcoxon rank-sum test, *P* = *2.2* × *10*^*–16*^) (Figure S10). *P. simium* samples obtained from human sources demonstrated the lowest overall nucleotide diversity (π range: 2.00 × 10^–6^ to 6.94 × 10^–4^), followed by similar nucleotide diversity in *P. simium* isolates from NHPs (π range: 1.40 × 10^–5^–6.86 × 10^–4^) (Wilcoxon rank-sum test, *P* > *0.01*) (Figure S11). In contrast, *P. vivax* samples from Brazilian districts had greater nucleotide diversity (Figure S11). Although, the sample size is small, this analysis supports the previous postulated hypothesis that *P. simium* infections of NHPs occurred due to a reverse-zoonotic spillover of *P. vivax* from humans^8^.

### Identification of mutations in P. vivax drug-resistance candidate orthologues

Given the strong genetic diversity observed in *P. vivax* populations from South America, we examined non-synonymous mutations in candidate drug-resistance genes within *P. simium* to assess whether similar variation is present. Non-synonymous mutations were identified in *P. simium* and *P. vivax* isolates (n = 392) across five genes of interest (Table S8). The DHFR N117S mutation was identified in *P. simium* (81.6%) and *P. vivax* from Mexico (95%) and elsewhere (15%), with N117T being present in Brazil (n = 1). Other mutations connected with drug resistance such as DHFR N50K, R58K, S116G, and I173L were not present in *P. simium* isolates. Only one mutation at DHFR N410S was unique to *P. simium*. Among five missense mutations in DHPS, only two were present in *P. simium* isolates: G383A (*P. simium*: 89.5%, *P. vivax* Brazil: 2.3%, *P. vivax* Mexico: 100%, other *P. vivax* 55.5%) and G439E present only in isolates from São Paulo at low frequency. Previously observed MDR1 L1076F and M958T mutations were not present in *P. simium* isolates but were seen with the expected high frequency in other South American *P. vivax* infections*.* A previously unreported MDR1 S698G mutation, found outside of ABC transporter domain, was identified in both *P. simium* (73.7%) and at high frequency in other *P. vivax* isolates. Additionally, three MDR1 mutations (T723S (13.2%), H681R (2.6%), L18M (2.6%)) were only present in *P. simium* genomes. There were two additional mutations unique to *P. simium* not observed in *P. vivax* from South America, specifically in Kelch13 (K424N, 23.7%) and CRT (E56Q, 2.6%).

### Known and novel structural variations in population isolates

**Fig. 3:**
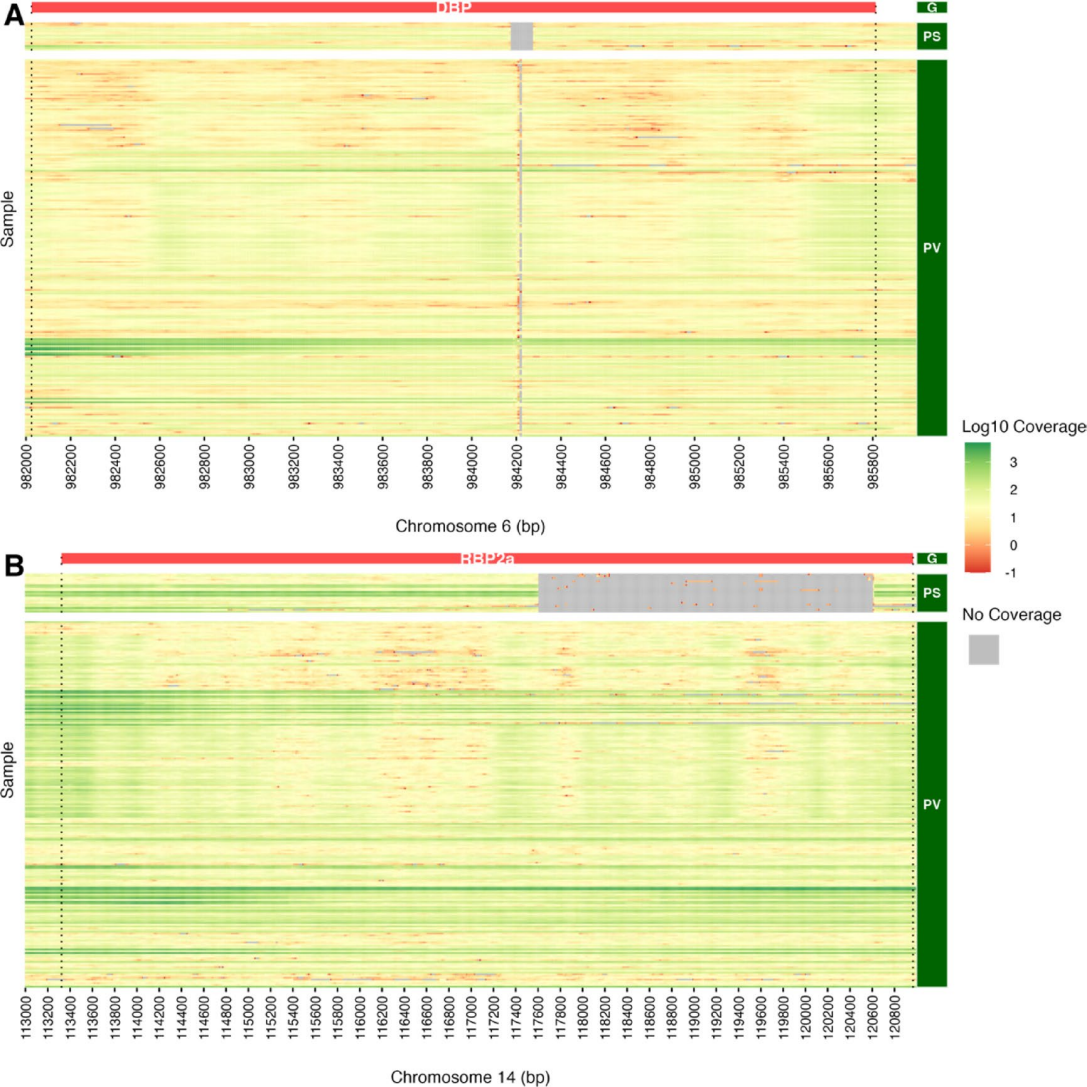
Deletion profiling with window-based (10 bp) coverage analysis for DBP (chromosome 6) and RBP2a (chromosome 14) applied to 392 isolates. Heatmap columns represent 10 bp genomic segments across each gene, rows represent individual samples, and the colour scale represents log10 coverage, with gray indicating 0 coverage. Samples are grouped by species. Additionally, only isolates with an average coverage greater than 5X across the inspected gene were included in the visualisation. (**A**) Duffy binding protein (DBP) and; (**B**) Reticulocyte-Binding Protein 2a (RBP2a).

Protein alterations revealed from the comparative *P. simium* Ps_SãoPaulo and *P. vivax* PvP01 genome analysis were assessed across the isolate data based on structural variant discovery by integrated paired-end and split-read analysis (see Methods). We report on 242 structural variants (195 deletions; 28 inversions; 15 insertions and four duplications) of which 114 were seen uniquely in *P. simium* and 26% of them were identified in genes of interest (Table S9). A deletion in DBP was present in both *P. simium* and *P. vivax* but at varying genomic breakpoints. The deletion is approximately 99 amino acids longer in *P. simium* (frequency 34%) than in *P. vivax* (frequency 21%). The deletion in RBP2 was solely unique to *P. simium* and observed in 63% of isolates (Fig. 3). Modifications in CSP (*P. simium* 31%, *P. vivax* ~ 10%) and CTRP (*P. simium* 34%, *P. vivax* 13%) had almost identical breakpoints in *P. simium* and *P. vivax* isolates. Remaining indels identified on protein level within genes of interest were confirmed by genomic analysis with identified inversions in MAEBL and TSR3 both at 10% frequency, a duplication overlapping MSP3.1 and MSP3.2 seen in 13% of samples, a deletion in TRAG13 (42%), and a small deletion and inversion in TRAG12 at 47% and 10%, respectively (Figure S12). Genomic analysis revealed additional deletions in PRP24 (45%), ARV1 (37%), DHX57 (37%), GCbeta (37%), and LISP2 (37%) solely unique to *P. simium* (Figure S13)*.* Several indels, identified outside of genes of interest, had high frequencies in *P. simium,* but similar breakpoints at lower frequencies in *P. vivax* species (Figure S14).

### Genomic regions under positive selection

To investigate recent positive selection in *P. simium* from Brazil compared to other South American *P. vivax* populations, we applied two complementary haplotype-based methods: integrated haplotype score (iHS) and cross-population extended haplotype homozygosity (XP-EHH). The iHS statistic detects evidence of recent selection within a population by measuring the decay of haplotype homozygosity around a given SNP, comparing the extended haplotypes carrying the ancestral and derived alleles. A high absolute iHS value indicates that one allele has risen rapidly in frequency, leaving a long-range haplotype, consistent with a recent selective sweep. In contrast, XP-EHH compares haplotype homozygosity between two populations to identify alleles that have become fixed or nearly fixed in one population but not the other. This makes it particularly useful for detecting selection events that have occurred in *P. simium* but not in *P. vivax*, or vice versa. Together, these methods provide complementary insights into recent or ongoing adaptation, allowing us to identify candidate loci under selection that may reflect host-switching events, environmental adaptation, or immune evasion specific to *P. simium* in Brazil.

For the *P. simium* samples obtained from Brazil, a combined total of 38 sites were potentially undergoing positive selection (iHS) (Table S10, Figure S15). Some sites were observed in known genes, including ATP4, LISP1, and MTIP. We also searched for gene products in genomic regions undergoing positive selection, which included proteins of unknown function, a signal recognition protein (SRP68), and genes involved in transcription and translation (Table S11). Due to the small sample size, a − log10[1–2 | ΦiHS–0.5 |] threshold of 2.5 was also used to identify 19 unique sites in *P. simium* considered to be undergoing selection (XP-EHH) (compared to South American *P. vivax*) (Table S12, Figures S16-19). Such sites were found to overlap between geographical comparators and were observed in similar genes, including *I2,* PVP01*_1142200* and PVP01*_0708700*. A single hit was also detected in *MND1* (Colombia). Gene products in genomic regions considered to be undergoing positive selection were also compared across populations (Table S13). Two regions on PvP01*_v1* chromosome 5 (1,010,000–1,040,000) and chromosome 10 (180,000–200,000) had a mean XP-EHH > 2.5 and were observed in the comparison between *P. simium* and *P. vivax* populations from Brazil. The majority of genes encoded products with unknown functions, but also included a *Plasmodium* exported protein (PHIST) with unknown function; putative glutamyl-tRNA(Gln) amidotransferase subunit A; putative DNA helicase MCM9; and phosphoenolpyruvate.

These findings suggest that *P. simium* is undergoing recent and potentially adaptive changes in response to unique selective pressures distinct from those acting on South American *P. vivax* populations. The identification of shared and population-specific signals may reflect evolutionary adaptation to different hosts or environments following the zoonotic transition. The presence of selection signals in both known and uncharacterised genes highlights the importance of further functional characterisation to better understand the molecular basis of *P. simium’s* divergence and adaptation.

### Comparison between P. simium isolates obtained from human and non-human primate sources

To investigate the genetic basis of host adaptation in *P. simium*, we performed a population differentiation analysis (F_ST_) between parasites isolated from humans and NHPs. This approach aims to identify genomic loci showing high divergence between the two host-associated groups, potentially reflecting selection pressures associated with adaptation to different host environments. Within the *P. simium* dataset, comprised of novel and previously published WGS data, 32 were obtained from *Homo sapiens* and six from *Alouatta guariba* (Brown Howler Monkey) (Table S6). Given the small sample size, analysis of selection was not possible. Therefore, we opted to compare these populations using F_ST_ to identify any SNPs that may be specific to *P. simium* isolates coming from each source. In total, 24 variants had high F_ST_ (> 0.85) (Table S14). Eighteen variants were observed exclusively in *P. simium* isolated from brown howler monkeys, including five intergenic variants, three intronic variants (PVP01_1109400, BPLP, PVP01_0813100 and CEP76), three synonymous mutations (PVP01_0813100 N629, CRMP1 C1544 and PVP01_1105700 S3105), and six nonsynonymous mutations (PVP01_1247800 S315C, PVP01_0824300 A148S, PVP01_1022500 A7418T, PVP01_1257400 F99I, PVP01_1270700 G10V and PVP01_1313300 G996D) (*P* < *0.01*). The remaining mutations with high F_ST_ were shared between *P. simium* isolated from humans and brown howler monkeys but found at low frequencies in human-derived samples. An intronic variant in SMC2 with F_ST_ of 0.86 (*P* < *0.01*) was found mostly in human samples (N = 18) rather than brown howler monkeys (N = 1). Such variants in candidate genes may be involved in host switching.

## Discussion

In recent years, there has been an emergence of malaria cases in humans in Brazil, particularly in the Atlantic Forest, which are attributed to the NHP parasite *P. simium*. The close genetic similarity between *P. simium* and the human malaria parasite *P. vivax* complicates accurate identification and hinders effective monitoring of its spread. Here, we present a much-needed draft genome of *P. simium* (*‘*Ps_SãoPaulo*’*) along with whole-genome sequencing data from 12 novel isolates, providing a valuable resource for future studies of its genomic diversity. A previous assembly exists, but we demonstrate that a hybrid approach combining Illumina and ONT sequencing yields a significantly more complete and accurate reference genome^8^. While the assembly is not an evenly balanced mosaic of the three samples, the strong contribution from ERR10301330 provides a robust and high-quality backbone for the genome complemented by unique sequences from the other samples that add valuable genomic coverage to the final assembly. Comparative analysis of the Ps_SãoPaulo draft genome and *P. vivax* P01 shows a high level of conservation between most of the proteins. The Ps_SãoPaulo genome contains *P. simium*-specific mutations in drug resistance genes, two SNPs in mitochondrial sequence, and previously detected deletions, including in *DBP* and *RBP2a* genes^8^. The absence of drug-resistance markers in *P. simium* may reflect the limited selective pressure exerted on these parasites. However, we observed small deletions in *msp3.1, msp3.2* and *csp,* which are loci proposed as potential vaccine candidates for malaria^17^. Alterations in parasite surface proteins (such as RBP, MSP, CSP, and AMA1) and host-specific receptors have played key roles in the adaptation of other NHP malaria parasites, including *P. malariae*, *P. brasilianum*, *P. knowlesi*, and *P. praefalciparum*. The identification of *P. simium* specific markers will assist with future diagnosis and species identification. Additionally, sequencing new isolates from Southeast Brazil, originating from both human and NHP infections, would help validate species-specific polymorphisms, enhance poorly resolved regions of the reference genome (e.g., subtelomeric and apicoplast regions), and provide deeper insights into parasite evolution. Although the presented *P. simium* reference genome is notably shorter than *P. vivax* P01, it is of higher quality than existing genome assemblies for this species. This is likely due to poor representation of sub-telomeric regions, including *vir* genes (~ 1000 genes present in *P. vivax*). Future updates to the *P. simium* genome assembly should strive to improve sub-telomeric regions of the genome, which are typically difficult to assemble due to their repetitive nature, high variability and structural complexity. Greater genome coverage of these regions, combined with the use of the Latin American PvPAM reference genome—which has superior subtelomeric contiguity—as a guide, could enhance future genome assemblies^14^.

Our data support the current hypothesis that *P. simium* resulted from a *P. vivax* transfer from humans to local monkey species on the American continent, as demonstrated by the low nucleotide diversity across *P. simium* isolates^8^. Whilst determining the origin and date of divergence of *P. simium* samples has not been possible in this study, glimpses into *P. simium* genome evolution were made through comparative and population genetic analyses. We identify 114 structural variants that are unique to *P. simium,* in addition to non-synonymous mutations in candidate genes implicated in drug resistance. While a larger sample size would enhance the power of these analyses, we were nevertheless able to identify genomic regions and gene products likely under positive selection in the *P. simium* population, particularly those involved in hepatocyte egress, red blood cell invasion, and key processes such as transcription and translation. In the future, the inclusion of more samples may generate greater insight into the population genetics of *P. simium,* but our results provide an initial indication of potential adaptive mechanisms. Additional characterisation of genes encoding proteins with unknown functions may also improve understanding of the mechanisms involved. Although there is currently no in vitro culture system for *P. simium* (or *P. vivax*), functional validation of genetic findings could be pursued through orthologous gene replacement in *P. knowlesi*, which is amenable to laboratory culture and genetic manipulation^18^.

To investigate the evolutionary relationship between *P. simium* and *P. vivax* and explore genome-wide patterns of variation, we performed comparative genomic analyses using the well-characterised PvP01 reference genome. Although the Latin American PvPAM genome is geographically relevant, the PvP01 reference genome is well-established and has more comprehensive annotation, making it more suitable for comparative analyses. However, the PvPAM reference genome could be utilised in the future to complement PvP01 for more geographically relevant comparative genomics^14^. Although reads from *P. simium* infections were mapped to the *P. vivax* reference to enable direct comparisons with *P. vivax* populations, mapping to the newly available *P. simium* genome could also capture additional variable sites, representing an important avenue for future analyses. However, by comparing genomes mapped to PvP01, the mechanisms that drive host switching and host adaptation that are key to understanding the potential of *P. simium* to infect humans could be investigated. The mutations observed in RBPa and DBP provide a logical explanation for adaptation to NHPs, given their established role in reticulocyte binding and invasion, but additional mechanisms are likely to be involved^8,19^. Even so, a key question remains as to how *P. simium* can retain the ability to infect humans. Although sample sizes for samples obtained from NHPs are limited, comparison between these populations (F_ST_) generated variants in genes of interest. This included mutations in SMC2 (Structural Maintenance of Chromosomes 2) and CEP76 (Centrosomal Protein 76), which are involved in chromosome organisation and formation, respectively; PBLP (Plasmodium Berghei Ligand Protein), which interacts with the host immune system and plays a role in adhesion and signalling pathways between parasite and host cells; CRMP1 (Collapsin Response Mediator Protein 1), which is implicated in host cell interaction and may affect the parasite’s ability to invade and migrate; and, finally, PUF1 (Pumilio Family RNA-Binding Protein 1) which regulates gene expression. Each of these genes plays a role in essential biological processes required for the parasite’s survival, growth, and reproduction, including mechanisms of immune evasion and red blood cell invasion. Such mutations could underpin the evolutionary flexibility of *P. simium* and its capability to adapt to changes in host environments and defenses. As *P. simium* is thought to have originated from *P. vivax*-like parasites infecting humans before undergoing a host switch to NHPs in the Atlantic Forest, the recent re-emergence of *P. simium* infections in humans raises the possibility of reverse zoonosis. Under this scenario, certain genetic changes may facilitate the parasite’s ability to re-infect and adapt to the human host. Identifying highly differentiated variants, such as those described, between human- and NHP-derived *P. simium* therefore reveal candidate genes involved in this re-adaptation process and help uncover molecular mechanisms enabling cross-species transmission. Further research involving larger sample sizes is needed to deepen our understanding of the mechanisms driving *P. simium* outbreaks in humans.

In lieu of such studies, our results showcase the utility of ONT sequencing and de novo assembly to produce a draft reference genome of *P. simium,* an important malaria parasite in Brazil with a complex evolutionary history. The creation of the reference genome provides essential insights into the structure, organisation and variation of the *P. simium* genome to guide future research. We also detect regions of interest within the *P. simium* genome through comparative genomics and population genetic analysis that may not only highlight regions under positive selection but also serve as future markers for genome-based diagnosis and epidemiological surveillance.

## Methods

### Sample selection and DNA sequencing

Twelve *P. simium* DNA samples were extracted from blood samples obtained from the Malaria Reference Laboratory, Health Department, São Paulo, Brazil, from patients who were diagnosed with malaria between 2013 and 2020. Informed consent was obtained from all participants (Ethical approval CAAE 67,855,517.1.0000.0064). To enrich *Plasmodium* DNA, a *P. vivax* spp. selective whole genome amplification (SWGA) primer set was used^20^. After SWGA, samples were purified using a 1:1 ratio of AMPure XP beads (Beckman-Coulter), following the manufacturer’s instructions and quantified via a Qubit™ dsDNA Quantification Assay Kit (Thermo Fisher Scientific Inc.). Short read sequencing (paired end 150 bp reads) of the DNA samples (n = 12; Table S1) was performed on an Illumina NovaSeq 6000 platform by The Applied Genome Centre, LSHTM (genomics.lshtm.ac.uk). For the three isolates selected to create the “Ps_SãoPaulo” reference genome, long-read sequencing data was obtained in two rounds using Oxford Nanopore Technologies (ONT) MinION at The Applied Genome Centre, LSHTM. The three isolates were first prepared for sequencing using the ONT LSK-109 and EXP-NBD104 barcoding kit (ONT) as per the manufacturer’s instructions. To select fragments of greater mass during the library preparation procedure, LSB buffer (ONT) was used during magnetic bead clean-up, and 120 ng of DNA library was loaded onto the R10 flow cell for sequencing with adaptive sampling rejecting reads associated with the human genome (GRCh38). Following SWGA enrichment and T7 endonuclease (NEB-M0302S) treatment (New England Biolabs), as per the manufacturer’s protocol (WAL_9070_v109_revQ_14Aug2019), samples were again prepared following the same methodology and sequenced using an R10 flow cell (ONT). All resulting fast5 files were base-called using Bonito (ONT) (models: dna_r10.3 and dna_r10.4), and the reads generated were subsequently trimmed and demultiplexed using Porechop software (v0.2.4). All sample-specific long-read data obtained were subsequently pooled for de novo assembly. In addition, 380 WGS for publicly available *P. vivax* and *P. simium* samples were incorporated in this study (Table S6).

### Quality control and reference genome assembly

Alignment of Illumina short reads was performed with bwa-mem (v0.7.17-r1188; default options), and minimap2 (v2.24-r1122) for ONT long reads against the *P. vivax* PvP01 (v2) reference genome (sourced from PlasmoDB release 59). Genome coverage was calculated using mosdepth (v0.3.3)^21^. Scrubbing to remove chimeric reads was performed for ONT reads using yacrd software (v1.0.0; options -c 4 -n 0.4 scrubb)^22^. Kraken2 software was used to classify taxonomic reads, and KrakenTools (https://github.com/jenniferlu717/KrakenTools; options –fastq-output –taxid –exclude) was applied to remove non-Plasmodium contaminants^23^. De novo hybrid assembly was performed using SPAdes software (v3.15.4; options –nanopore)^24^. Contaminated contigs were identified using the Blobtools (v1.1.1) suite^25^. The quality of the assembly was assessed using QUAST (v5.0.2) and abyss-fac (v2.3.4) software tools, which estimate the total size (bp), number of contigs, longest contig, N50, and similar metrics^26,27^. The distribution of gap sizes per million base pairs was calculated. Gene completeness was estimated using BUSCO software (v5.2.1; options -l plasmodium_odb10 -m geno –long)^28^. The Ps_SãoPaulo genome is available via the European Nucleotide Archive (GCA_965178745.1).

To evaluate the contribution of each sample to the final assembly, we analysed coverage depth and reference allele distribution across the genome. Per-sample coverage was calculated using mapped read data (minimap2 v2.24-r1122), and genomic regions exhibiting coverage greater than 2.5-fold from a single sample were identified^29^. Additionally, single-sample reference alleles were detected by examining positions where the reference allele was uniquely observed in only one sample. These analyses enabled quantification of sample-specific contributions to the assembly and identification of regions dominated by individual samples.

BLASTn and seqkit (v2.1.0) tools were used to extract contigs with mitochondrial sequences from assembly based on nucleotide homology that were investigated visually with Jalview^30–32^. Contig polishing was performed by PILON (v1.24; options-fix-all-vcf) and SSPACE (v2; options-t 0-o 10-m 25-r 0.9) (https://github.com/nsoranzo/sspace_basic) software tools using Illumina short-read data^33,34^. This process was applied iteratively, where raw reads after contamination removal were mapped with bwa-mem (v0.7.17-r1188) onto the assembly, followed by polishing with PILON and contig extension with SSPACE^35^. Extended contigs were used to form scaffolds with RagTag software with correct and scaffold commands (v2.1.0; default options)^36^, and subsequent gap closing was performed with Gapfiller (v1.11; options-m 10-o 30-r 0.8-n 10-d 100,000-t 10-i 10)^37^. Polished and scaffolded contigs were annotated using the online Companion suite^38^. The following options were selected: additional contiguation into pseudochromosomes, protein evidence, no transcript evidence, and annotation transfer with RATT from PvP01v2 (similarity threshold 40% with maximum gene length 100 kb).

### Comparative genomics analyses

The *P. simium* reference was also compared to the short-read assembly of *P. simium* (GCA_913736665.1) using JupiterPlot (https://github.com/JustinChu/JupiterPlot) with default settings to confirm genome assembly consistency^8^. The synteny of chromosomal regions of the draft reference (Ps_SãoPaulo) to *P. vivax* PvP01 and other genomes was performed using nucmer software (v3.1, options*–maxgap* = *500–mincluster* = *100*)^11,39–41^*,* and visualised using the python-circos (v0.3.0) package. A maximum-likelihood tree of Ps_SãoPaulo and public *Plasmodium* mitochondria sequences (N = 75) was constructed. Mitochondria nucleotide sequences were first aligned using MAFFT (v7.525), and IQ-TREE (v2.3.6) was used to construct a maximum-likelihood tree (Options: -m MFP-bb 1000-alrt 1000-T AUTO), which was visualised using iTOL and rooted on the midpoint^42–44^. Structural variations (SVs) in proteins were detected by comparing predicted polypeptide sequences of *P. simium* Ps_SãoPaulo and *P. vivax* PvP01 orthologues using alignments generated by muscle software (v5.1)^45^. Alignments were compared in python to identify gaps and SNPs and calculate simple statistics such as Jaccard similarity scores (overlap between gene sets). In the situation when multiple polypeptide sequences were available for one protein, the most complete sequence was selected for calculations. Selected alignments were visualised with the ggmsa R package [52].

### Population genetics for *P. simium* and *P. vivax*

All raw reads including novel and public *P. simium* (n = 38) and *P. vivax* isolates (n = 354; Brazil 130, other South America 224) were pre-processed using an established pipeline (https://github.com/LSHTMPathogenSeqLab), which performs trimming (trimmomatic (v0.39)), mapping (bwa-mem (v 0.7.17-r1188)) and variant calling^35,46^. SNP and indel variants were called using the GATK suite (v4.1.4.1 Haplotype Caller) and were combined across the 382 isolates using the GenomicsDB and CombineVCFs commands^47^. Variants in sub-telomeric regions and with missingness > 20% were removed, leading to 123,902 high-quality SNPs. SnpEff software (v5.0; options–no-downstream–no-upstream–classic) was used to annotate mutations.

PLINK software (v1.90b6.18) was used to create a distance matrix and multidimensional scaling was performed on the distance matrix using R^48^. To evaluate modifications identified in polypeptide sequence analysis, we performed structural variant discovery with DELLY (v0.8.7; options *call–t ALL*) using aligned isolate data (N = 387; software had errors for five *P. vivax* samples)^49^. Delly outputs were parsed for all samples and collated separately based on species and indel type. Common breakpoints were identified by overlap analysis (50 bp search windows from each start and end position), and the frequency per species was calculated. Indels occurring in fewer than 10% of individuals within any species were excluded. Gene annotation was then performed on the remaining set. *P. simium* indels were reported if they overlapped with any of the genes of interest (Group A) or if the indel was seen in at least 35% of *P. simium* samples (Group B). Lastly, to check the uniqueness of indels, duplications or inversions, we compared identified breakpoints against *P. vivax* calls similarly by overlap analysis and reported it where available along with frequencies. Additionally, coverage around identified indels was inspected through window-based coverage analysis (10 bp) generated with mosdepth software for each sample.

Nucleotide diversity was estimated using VCFtools (v0.1.16) and calculated in 10 kb windows^50^. We performed a Wilcoxon rank-sum test (Mann–Whitney U test) to compare nucleotide diversity (π) between human-and NHP-derived *P. simium* samples, in addition to *P. vivax* samples. Further population genetics analysis was carried out using an established pipeline (https://github.com/LSHTMPathogenSeqLab/malaria-hub/tree/master/selection). F_WS_, a measure of within-host diversity, was used to assess clonality F_WS_ was calculated using the moimix package and allele frequency threshold of 0.001 (https://github.com/bahlolab/moimix).Only samples with F_WS_ > 0.95 (clonal infections) were retained for selection analyses, while polyclonal infections (F_WS_ ≤ 0.95) were excluded. Within population selection statistics (iHS) and cross-population (XP-EHH) were estimated using the rehh package in R (minor allele frequency > 0.01, F_WS_ > 0.95 thresholds)^51^. iHS is a measure of recent positive selection in a single population, whilst XP-EHH looks for recent positive selection occurring across two different populations. A (− log10[1–2 | ΦiHS–0.5 |]) threshold of 2.5 was used to identify regions considered to be under positive selection. Finally, F_ST_ analysis was used to identify population differentiation over genomic positions between samples obtained from *Homo sapiens* and *Alouatta guariba* using VCFtools v0.1.16^50^. A threshold of > 0.85 was used as a cut-off for high population differentiation. F_ST_ significance was assessed using a permutation test (10,000 permutations), in which population labels were randomly reassigned to generate a null distribution of Fst values from which empirical p-values were calculated.

## Supplementary Information

Below is the link to the electronic supplementary material.

Supplementary Material 1

Supplementary Material 2

## Data Availability

All samples newly sequenced are available on the European Nucleotide Archive and their individual run accessions have been provided (Illumina: PRJEB56411; Oxford Nanopore Technology: PRJEB81302). The *P. simium “* Ps_SãoPaulo *”* has been uploaded to a dedicated GitHub repository ( https://github.com/NinaMercedes/P_simium ) and to NCBI (GCA_050575225.1).
