# Supplementary material for: Novel zoonotic cases of Plasmodium simium from São Paulo with a reference genome of the Brazilian strain
Source: Sci Rep. 2025 Dec 12;15:43703. doi: 10.1038/s41598-025-27554-0 (PMC12700903; doi:10.1038/s41598-025-27554-0)
Supplement: Supplementary file 2 — Supplementary Material 2 [file 41598_2025_27554_MOESM2_ESM.docx]

**SUPPLEMENTARY FIGURES**

**Figure S1**

**Sequencing coverage across Illumina short read and Oxford Nanopore technology long read platforms.**

Average coverage across core (purple) and sub-telomeric (orange) genome regions for three selected isolates used in the draft genome assembly.


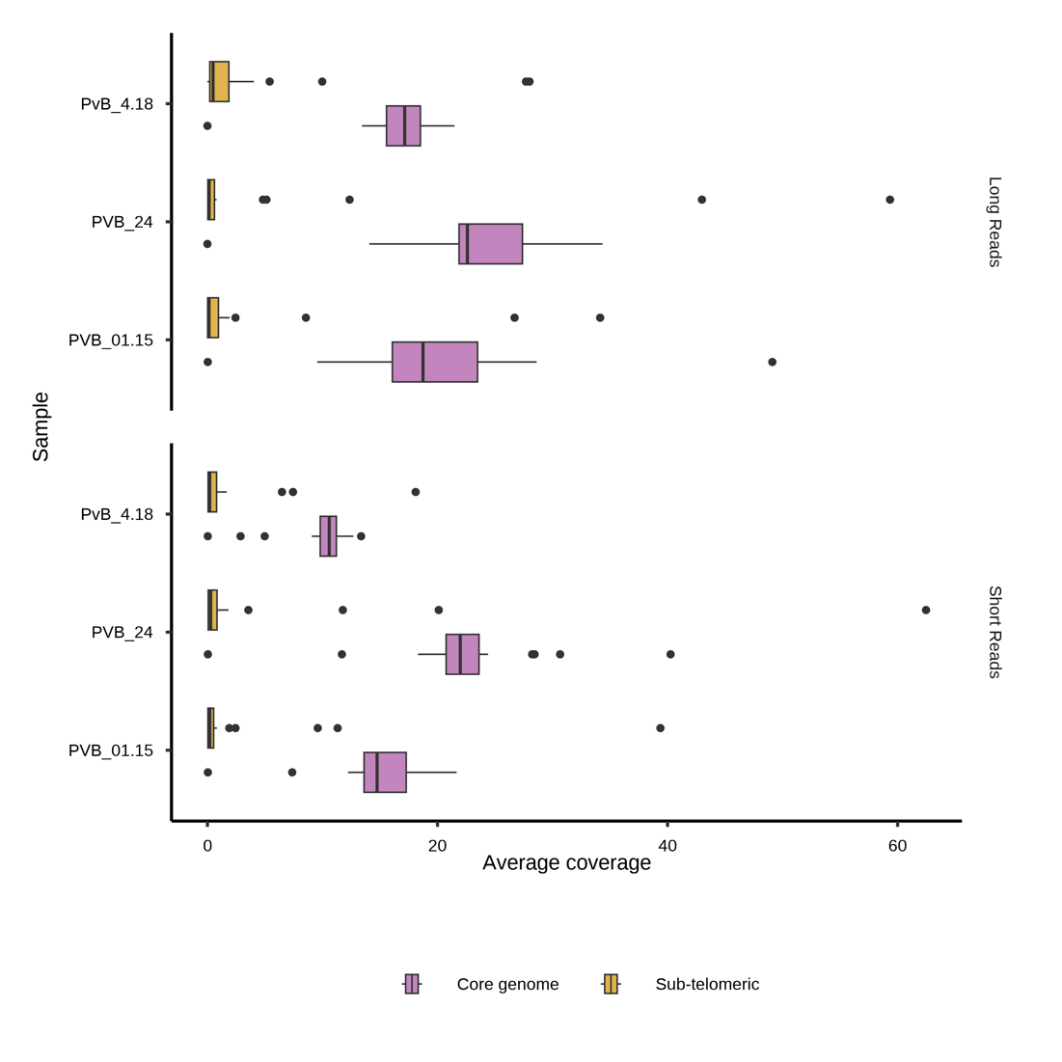


**Figure S2**

**Workflow for Hybrid assembly analysis**


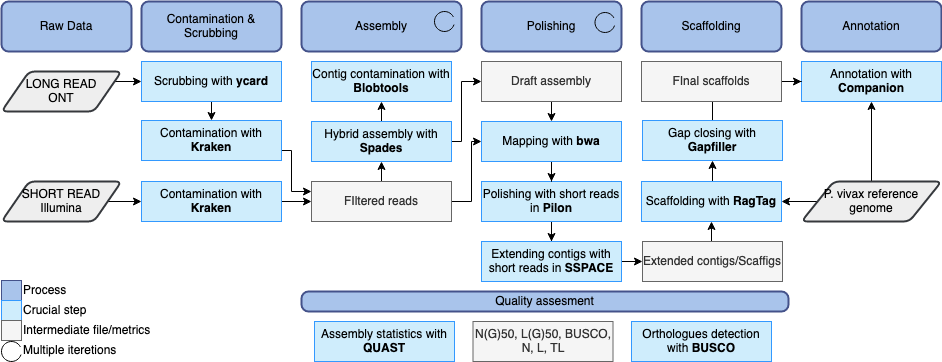


**Figure S3**

**Distribution of sample-specific SNPs across genomic windows to assess assembly composition.** The number of SNPs unique to each of the three *P. simium* samples used to generate the reference genome assembly, as well as those shared among samples (“mixed”), were counted within defined genomic windows. This analysis highlights regions of the genome with potential chimeric origin, indicating sequence segments that may derive from different samples or strains. The figure illustrates the genomic distribution and proportion of these SNP categories across the assembly.

**
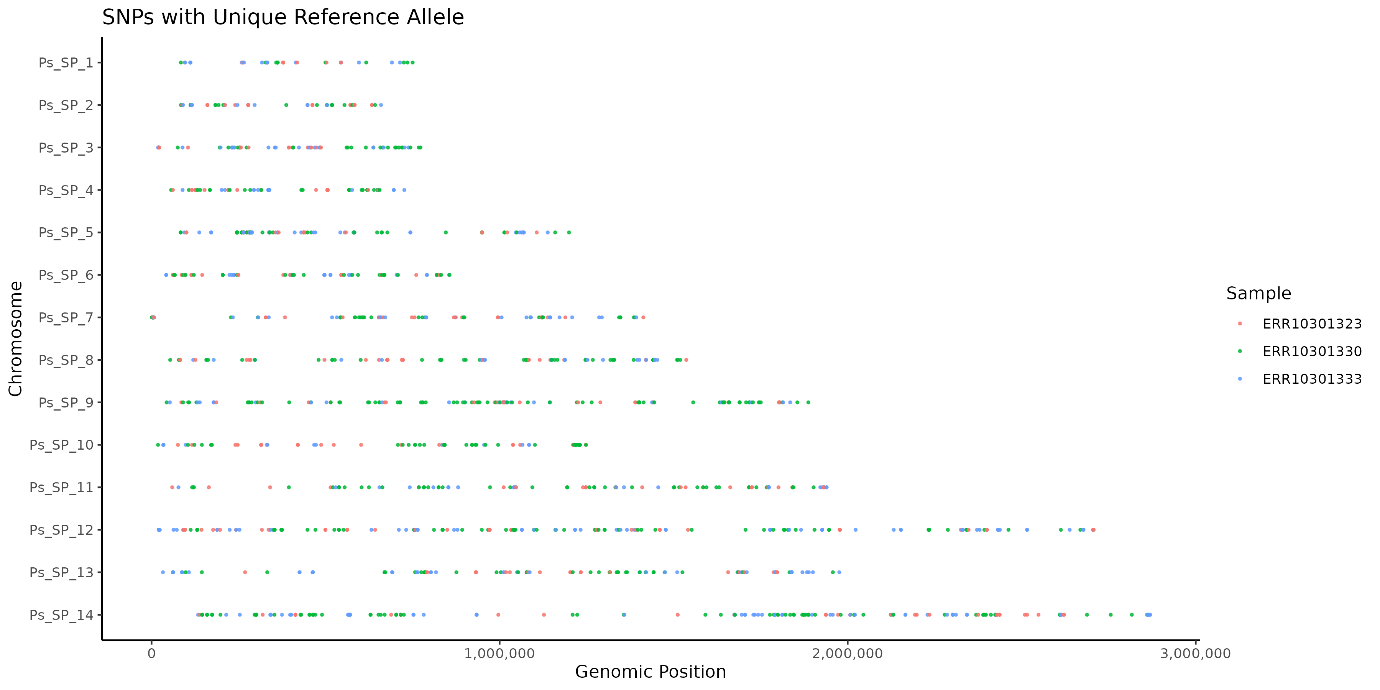
**

**Figure S4**

**Synteny of *P. simium*** Ps_SãoPaulo **genome and short-read assembly (Mourier *et al.*2021)**

JupiterPlot highlighting the synteny between *P. simium Ps_SãoPaulo* genome and short-read assembly (Mourier et al.2021). Colored chromosomes correspond to *P. simium Ps_SãoPaulo* genome. Black lines indicate ‘N’ (missing) values. The largest scaffolds of the short-read assembly (75% of the genome) are shown.


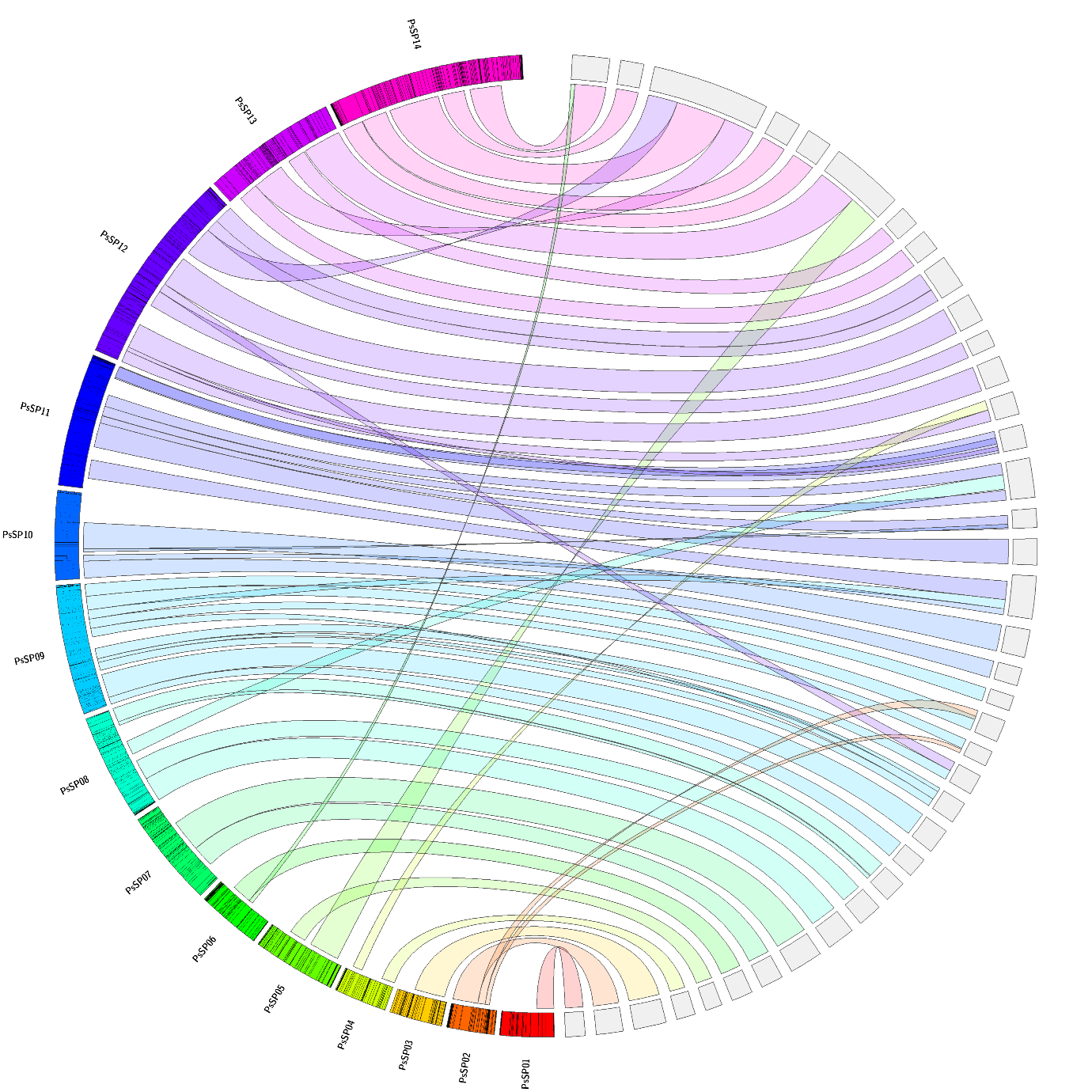


**Figure S5**

**BlobPlot summarising taxonomic annotation of assembly and long reads.**

Two-dimensional scatter plots with coverage and GC histograms generated with Blobtools for initial assembly of 4082 contigs to check contamination status. Legend reflects the taxonomic classification of sequences. **(A)** Sequences are represented by circles (diameter proportional to length) coloured by taxonomy and positioned by GC content (X-axis) and base coverage (Y-axis). **(B)** Coverage of mapped long reads to the initial assembly.


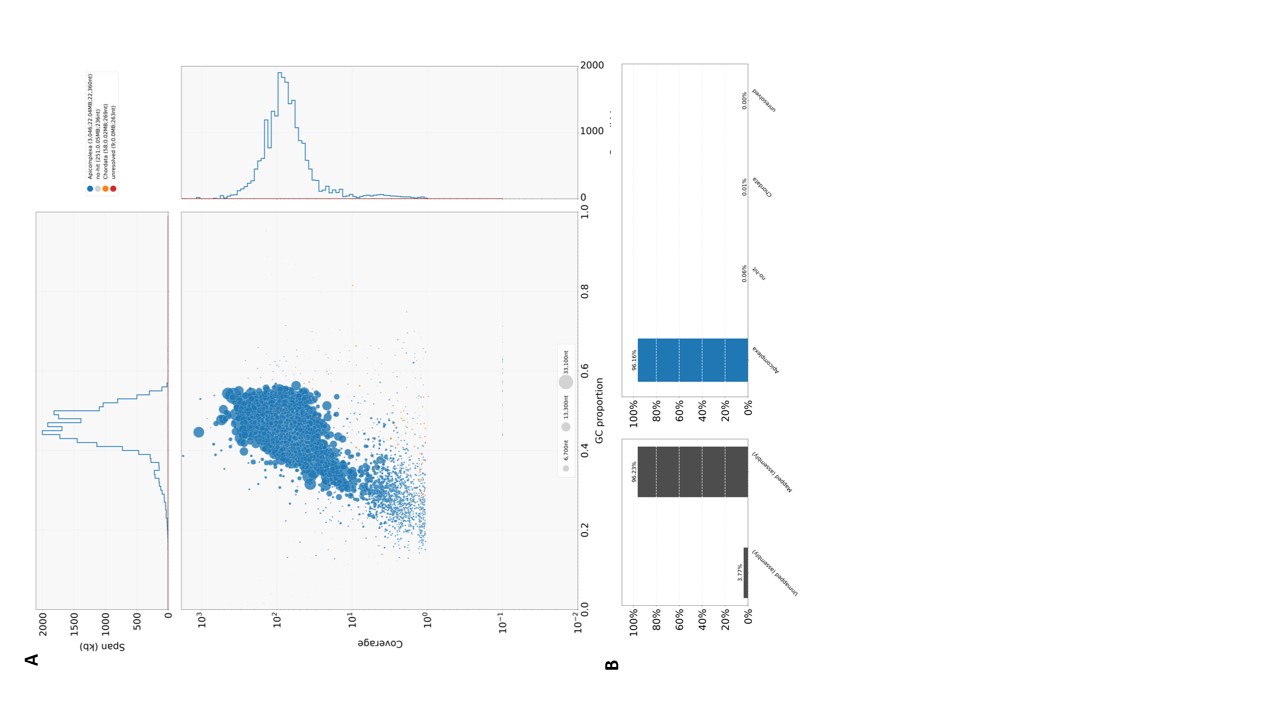


**Figure S6**

***P. simium* *Ps_SãoPaulo* gene annotation for each chromosome.**

Gene annotation coloured by the strand orientation and assigned to each chromosome. Contigs were combined and appended into Ps_SP_00 scaffold for visualisation purpose. Mitochondrial genes skipped for purpose of this visualisation.


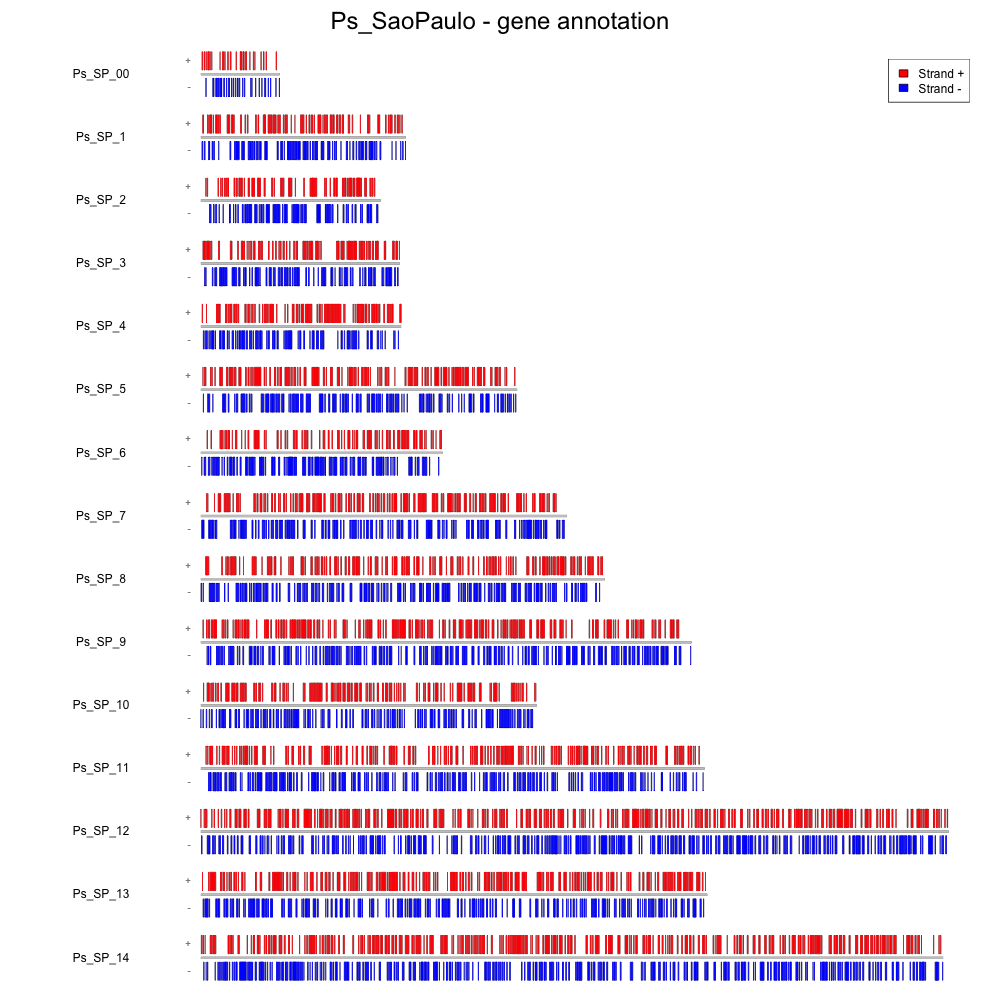


**Figure S7**

**Multiple protein sequence alignments of Duffy Binding Protein (DBP) for *P. simium* and closest orthologues across different species.**

Alignment between *P. simium* predicted protein (Ps_SãoPaulo_060028300), *P. vivax* P01 (PVP01_0623800), *P. vivax-like* (*P*VL_060021400), *P. vivax Sal I* (PVX_110810) and *P. cynomologi B* (PCYB_063270) confirmed that the larger deletion (91aa) is present only in *P. simium* reference. Shorter (9aa) deletions appear fixed in *P. vivax Sal I* and *P. cynomologi.*


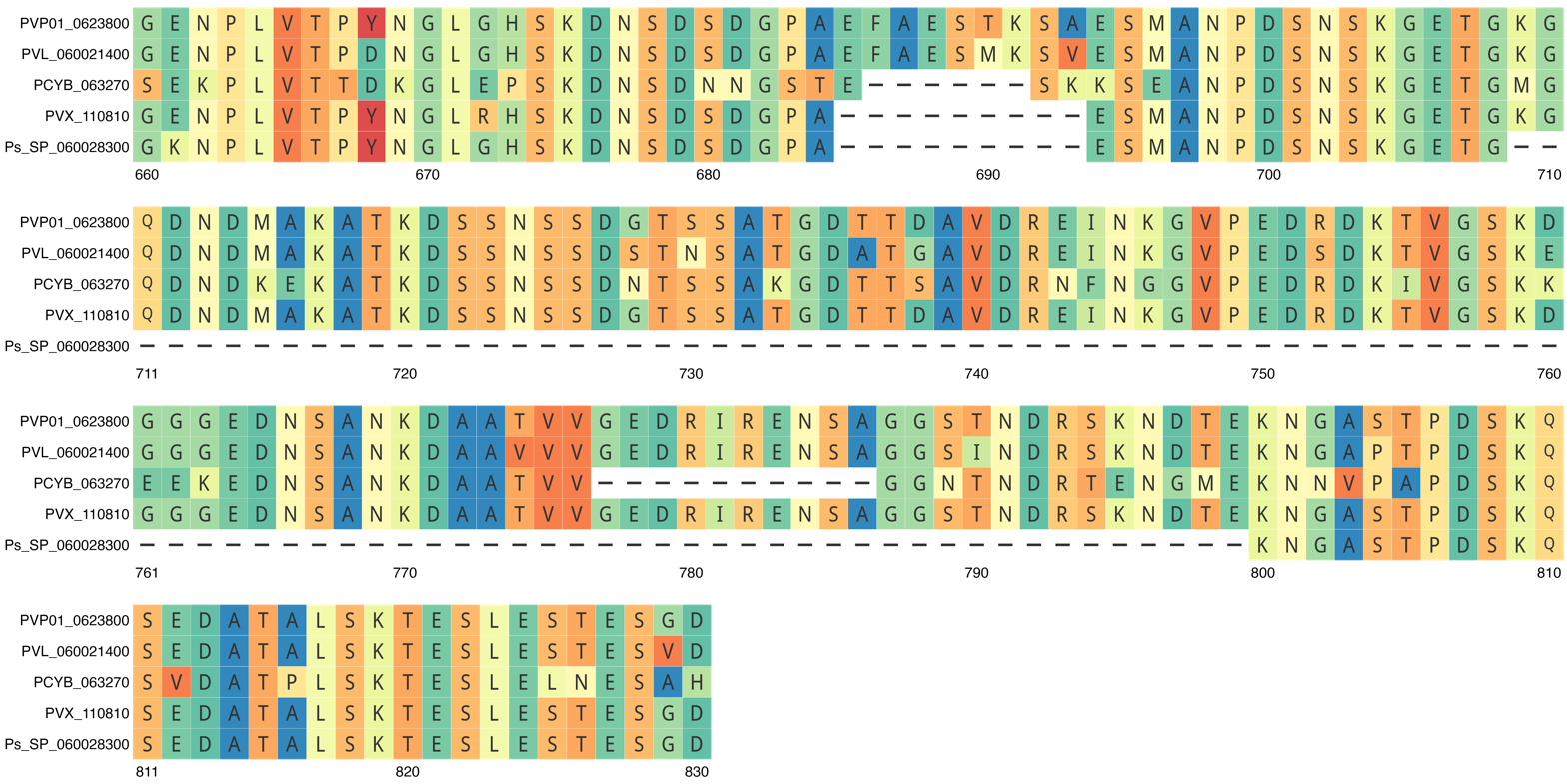


**Figure S8**

**Multiple protein sequence alignments of RBP2a for *P. simium* and closest orthologues across different species.**

Alignment of *P. simium* predicted protein (Ps_SãoPaulo_14000600), *P. vivax* P01 (PVP01_1402400), *P. vivax-like* (PVL_140006600) and *P. vivax Sal I* (PVX_121920) genes revealed that deletion is unique to *P. simium* only and additional insertion in *P. vivax-like* sequence.


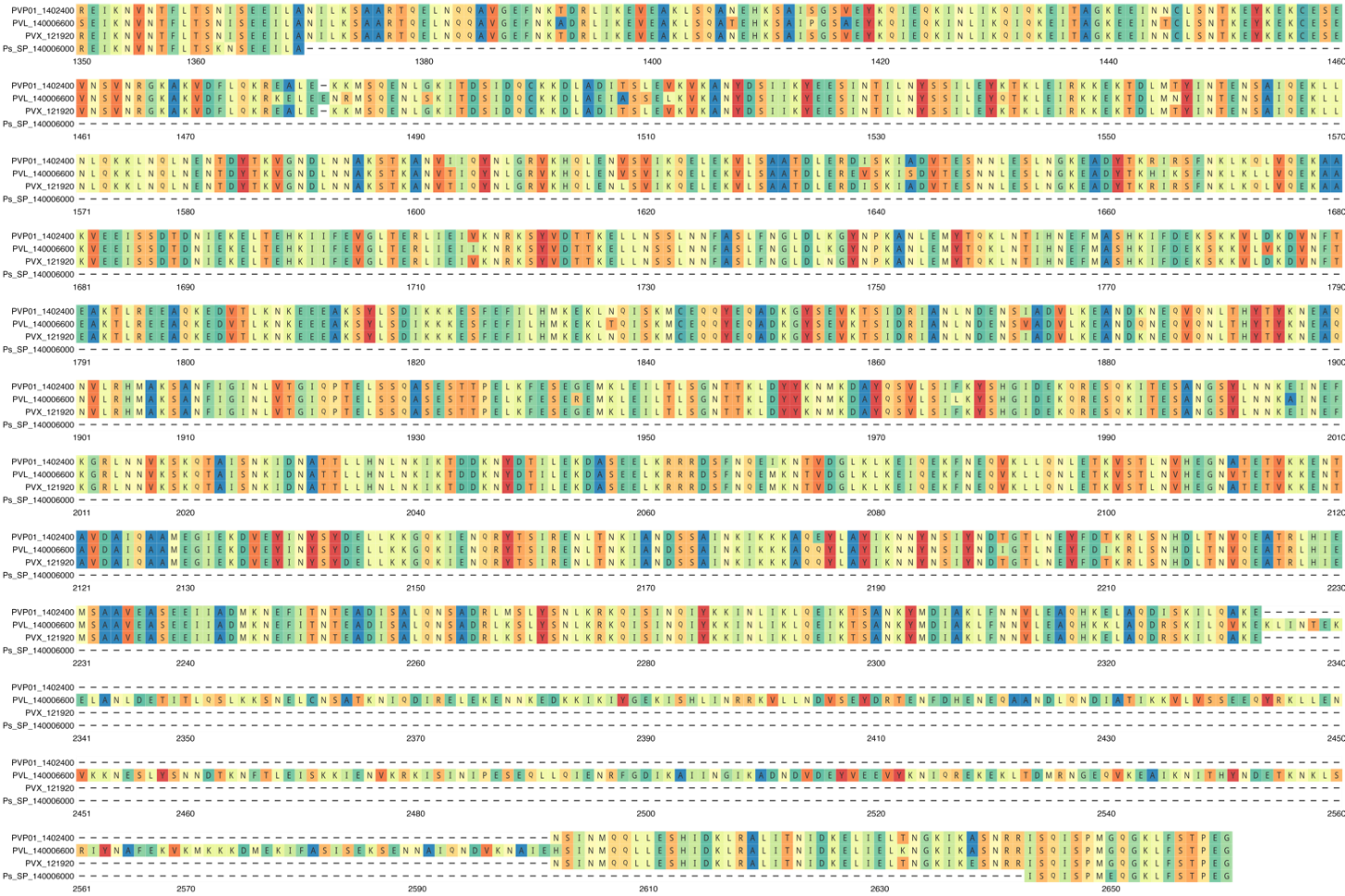


**Figure S9**

**Genotype frequency and average read depth for 4133 and 4467 mitochondrial positions across *P. simium* and *P. vivax* dataset.**

Mutations in mitochondrion in a dataset consisting of 392 samples with *P. vivax* and *P. simium* (fill=species). **(A)** Barplot of putative mitochondrial genotypes identified to be unique to *P. simium* genome on positions 4133 and 4467 (CG) and *P. vivax* (TA). **(B)** Read depth for putative mutations shows higher coverage in *P. vivax* species than *P. simium*.


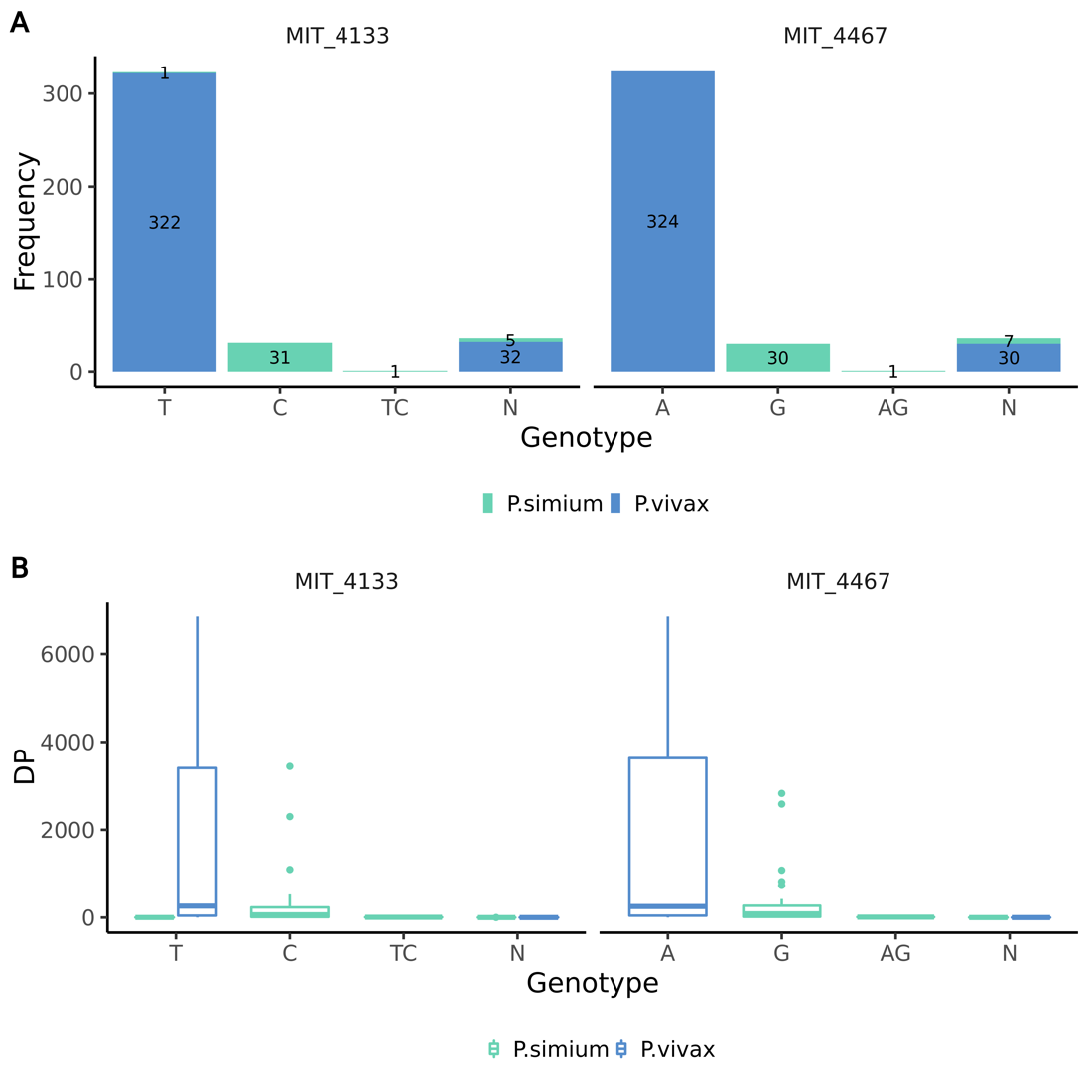


**Figure S10**

**Genome-wide windowed Nucleotide Diversity (10kb) of new *P. simium* isolates compared to *P. vivax* from Brazil (chromosomal plots)*.*** Nucleotide diversity measured in 10kb windows are shown for *P. simium* isolates obtained from human and non-human primates (NH) and compared to *P. vivax* samples from Brazilian districts. Nucleotide diversity (π) across each chromosome is shown.


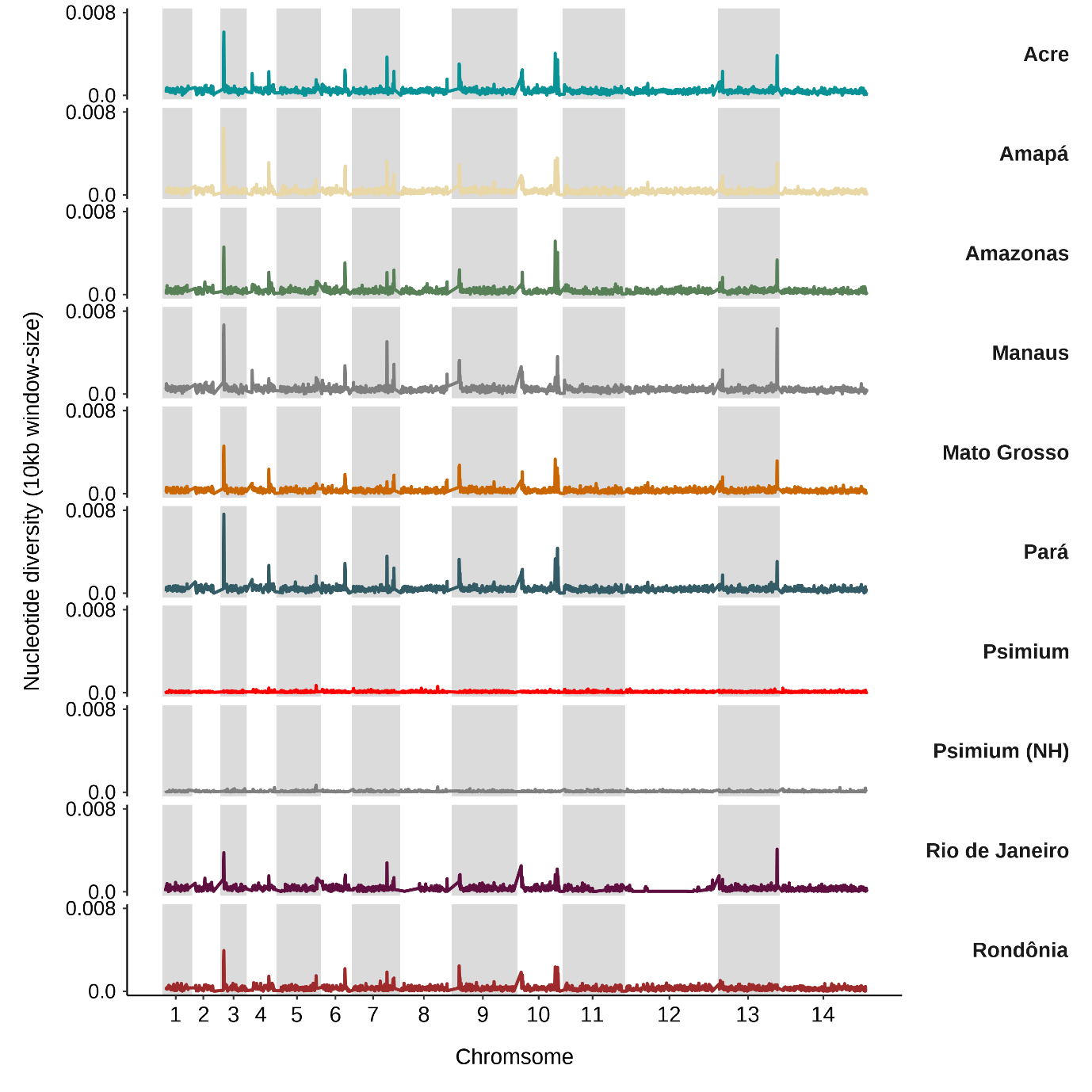


**Figure S11**

**Nucleotide Diversity of new *P. simium* isolates compared to *P. vivax* from Brazil (boxplots)*.*** Nucleotide diversity measured in 10kb windows are shown for *P. simium* isolates obtained from human and non-human primates (NH) and compared to *P. vivax* samples from Brazilian districts. Median, interquartile range and outlier values of nucleotide diversity (π) are shown.

**
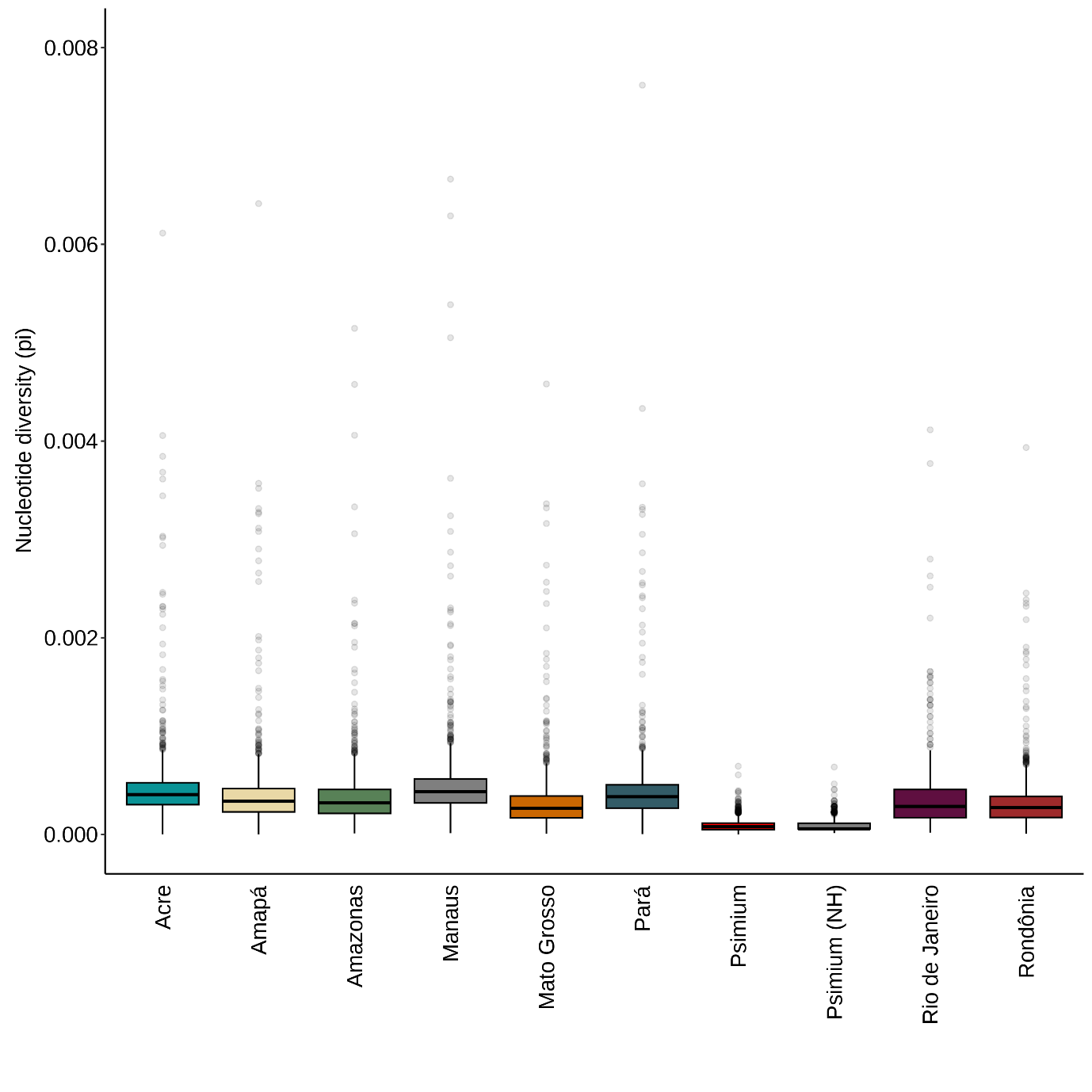
**

**Figure S12**

**Indel profiling with window-based (10bp) coverage analysis for genes identified in protein level comparisons presented across *P. simium* and *P. vivax* isolates*.***

Heatmap columns represent 10bp genomic segment across gene, rows represent individual sample and color scale represents log10 coverage, with gray indicating 0 coverage. Samples are grouped by species. Additionally, only isolates with average coverage higher than 5X across the inspected gene were included in the visualisation. **(A)** MAEBL on chromosome 9; **(B)** TRAG13 on chromosome 5; and **(C)** MSP3.1**;** and **(D)** MSP3.2 on chromosome 10.


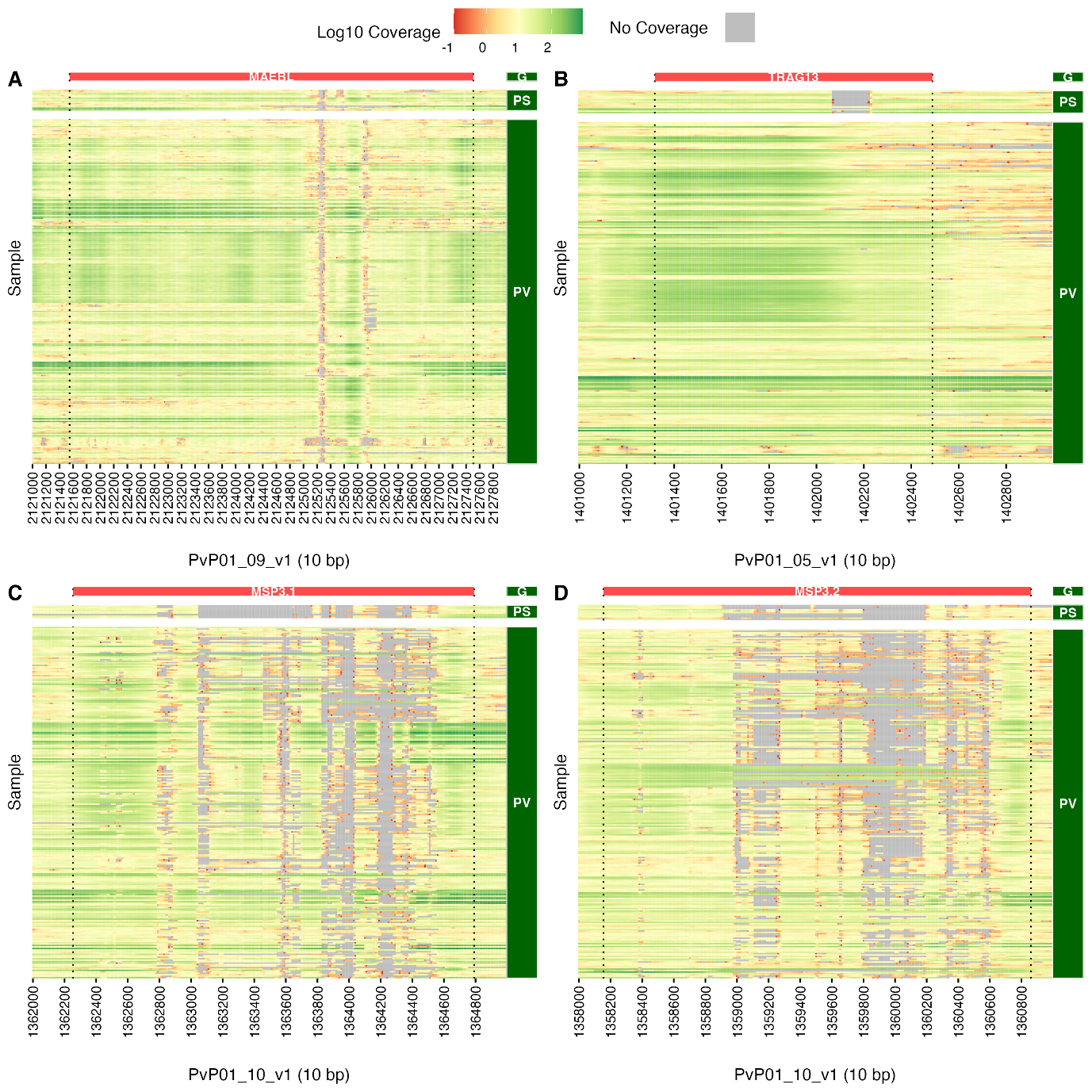


**Figure S13**

**Indel profiling with window-based (10bp) coverage analysis for genes identified in genomic level structural variants analysis unique to *P. simium* isolates presented across *P. simium* and *P. vivax* isolates**

Heatmap columns represent 10bp genomic segment across gene, rows represent individual sample and color scale represents log10 coverage, with gray indicating 0 coverage. Samples are grouped by species. Additionally, only isolates with average coverage higher than 5X across the inspected gene were included in the visualisation. **(A)** ARV1 and; **(B)** DHX57 both on chromosome 8; **(C)** LISP2 on chromosome 3**; (D)** PRP24 on chromosome 7.


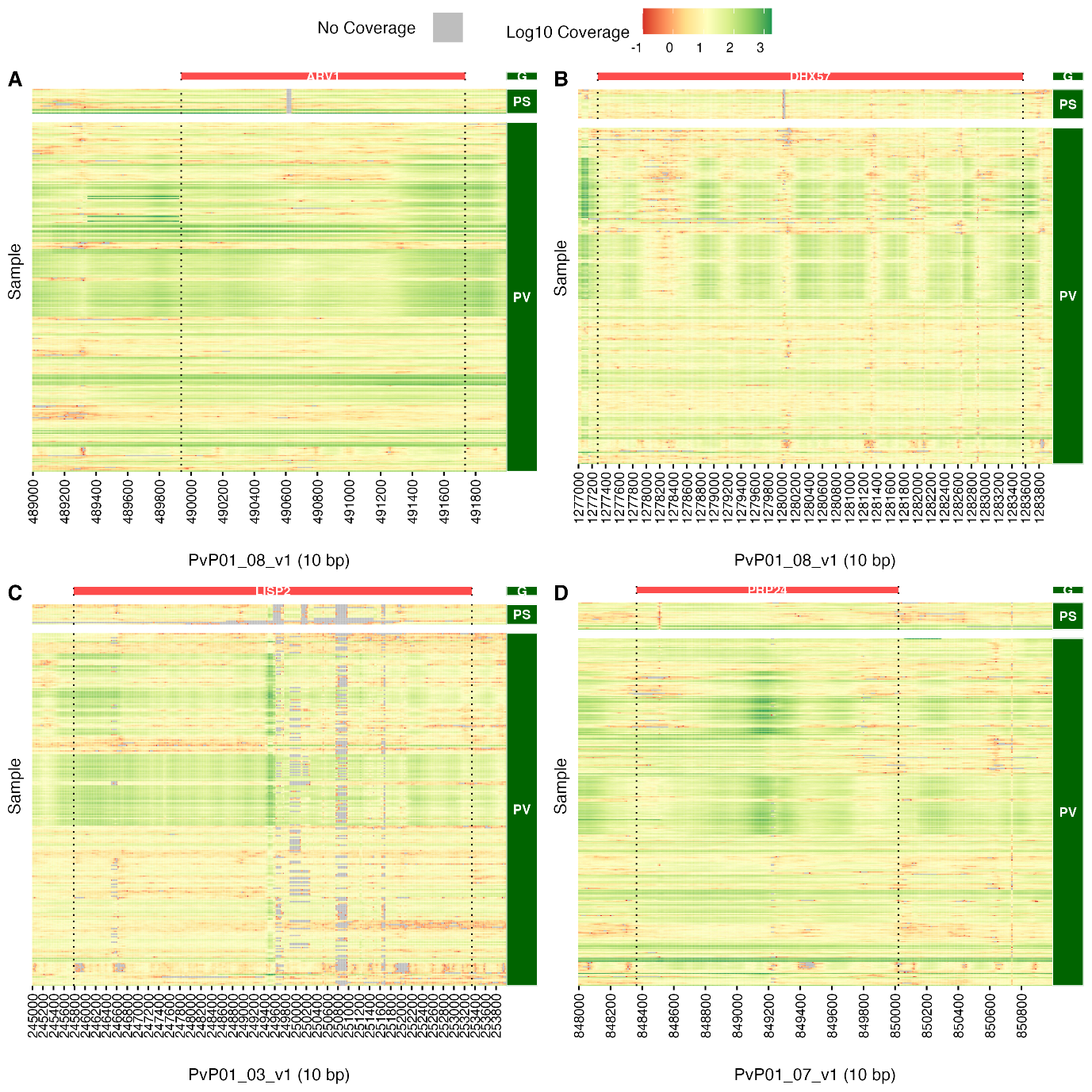


**Figure S14**

**Indel profiling with window-based (10bp) coverage analysis for genes identified in genomic level structural variant analysis conserved in *P. simium* and *P. vivax* isolates presented across *P. simium* and *P. vivax* isolates.**

Heatmap columns represent 10bp genomic segment across gene, rows represent individual sample and color scale represents log10 coverage, with gray indicating 0 coverage. Samples are grouped by species. Additionally, only isolates with average coverage higher than 5X across the inspected gene were included in the visualisation. **(A)** VPS15 on chromosome 5; **(B)** ARK2 on chromosome 8; and **(C)** UTP4**;** and **(D)** UBA1 both on chromosome 12.


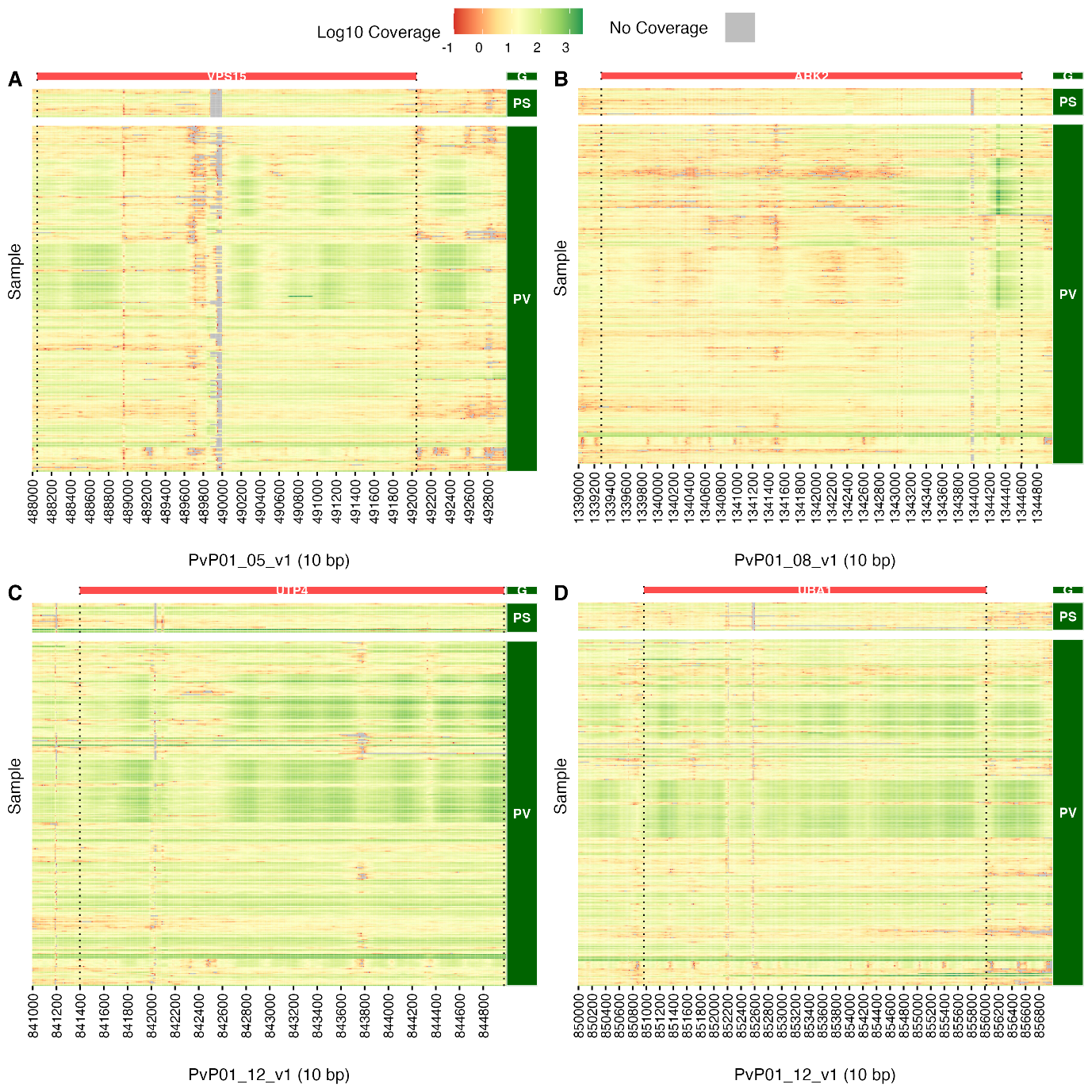


**Figure S15**

**Genome-wide scan for positive selection in *Plasmodium simium.***
(A) Manhattan plot displaying iHS values across the 14 nuclear chromosomes of *P. vivax* PVP01. Each dot represents an individual SNP, plotted according to its genomic position on the x-axis and the corresponding iHS value on the y-axis. Significant hits surpassing the predefined threshold are highlighted. (B) Histogram showing the distribution of p-values derived from the iHS analysis across the genome, illustrating the frequency of SNPs at different significance levels.

**
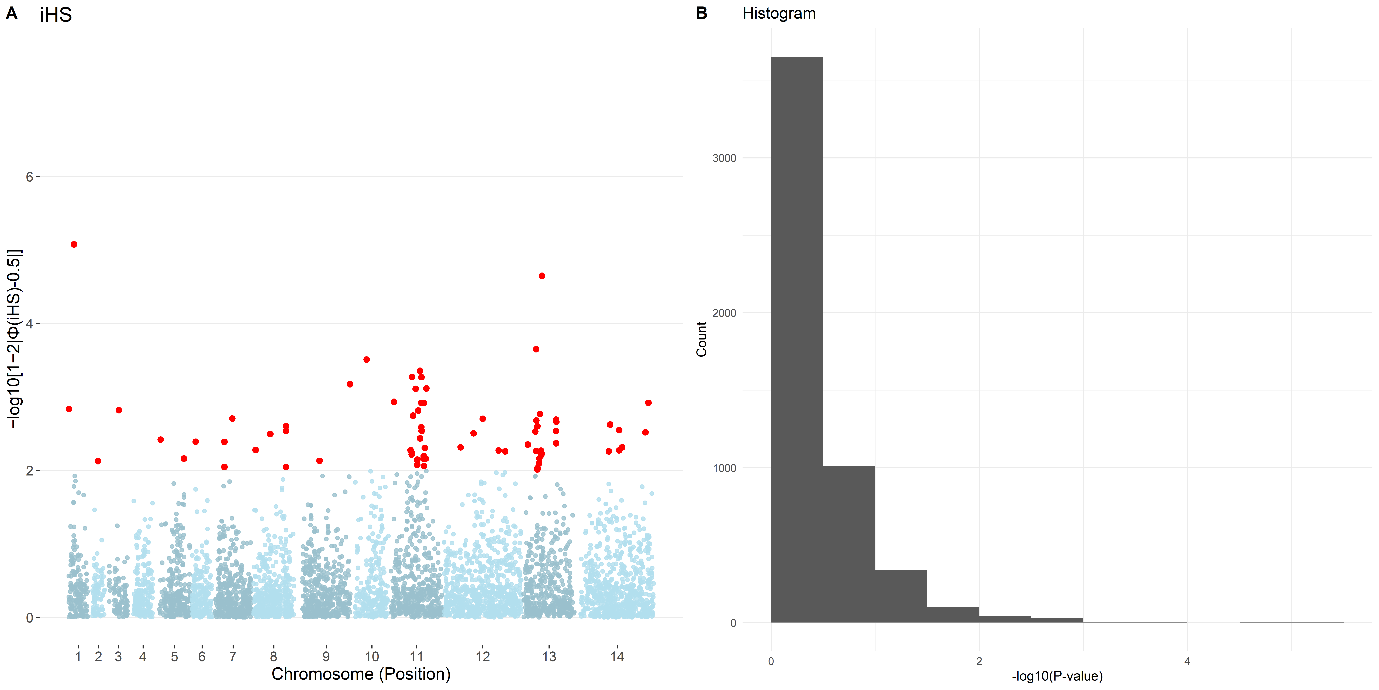
**

**Figure S16**

**Genome-wide XP-EHH analysis comparing *Plasmodium simium* and Brazilian *P. vivax*.**(A) Manhattan plot showing XP-EHH scores across the 14 nuclear chromosomes of *P. vivax* PVP01. Each point represents a SNP positioned by its genomic location on the x-axis and the XP-EHH score on the y-axis. SNPs with significant signals of recent positive selection in *P. simium* relative to Brazilian *P. vivax* are highlighted.
(B) Histogram depicting the distribution of p-values from the XP-EHH analysis across the genome, indicating the frequency of SNPs at varying significance levels.

**
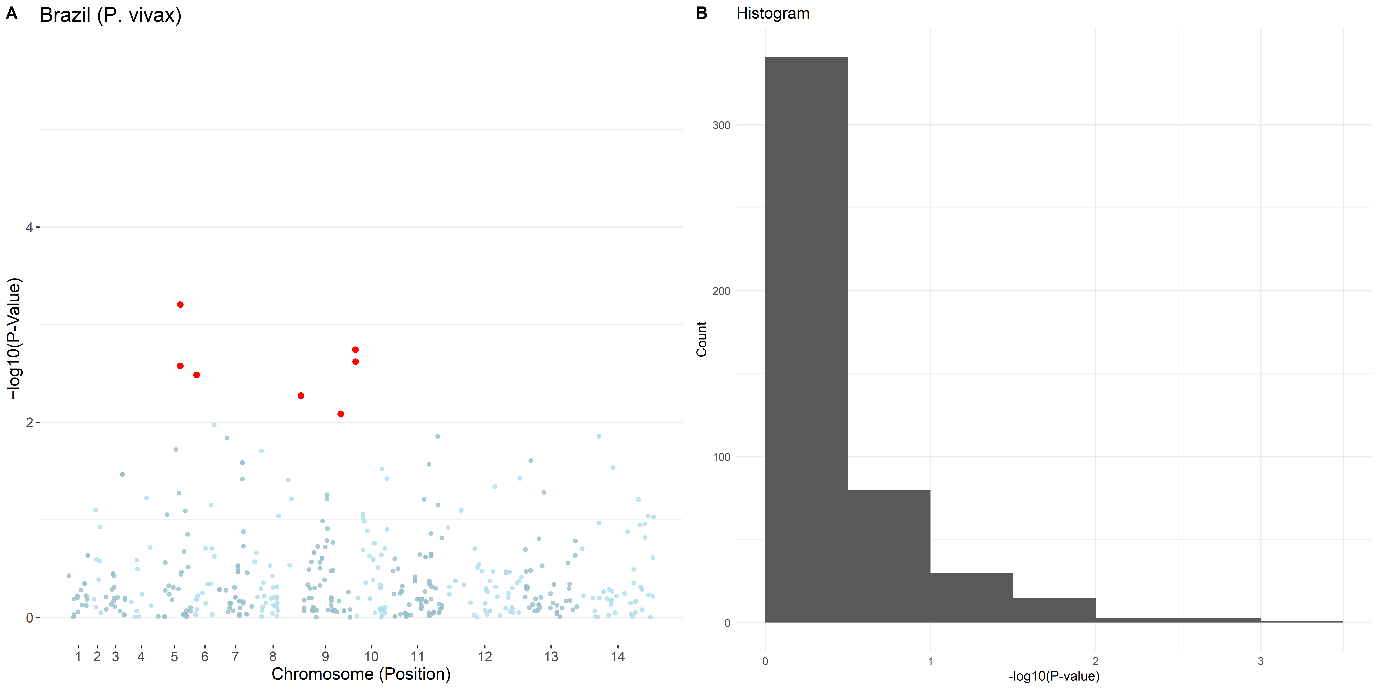
**

**Figure S17**

**Genome-wide XP-EHH analysis comparing *Plasmodium simium* and Colombian *P. vivax*.**(A) Manhattan plot showing XP-EHH scores across the 14 nuclear chromosomes of *P. vivax* PVP01. Each point represents a SNP positioned by its genomic location on the x-axis and the XP-EHH score on the y-axis. SNPs with significant signals of recent positive selection in *P. simium* relative to Colombian *P. vivax* are highlighted.
(B) Histogram depicting the distribution of p-values from the XP-EHH analysis across the genome, indicating the frequency of SNPs at varying significance levels. **
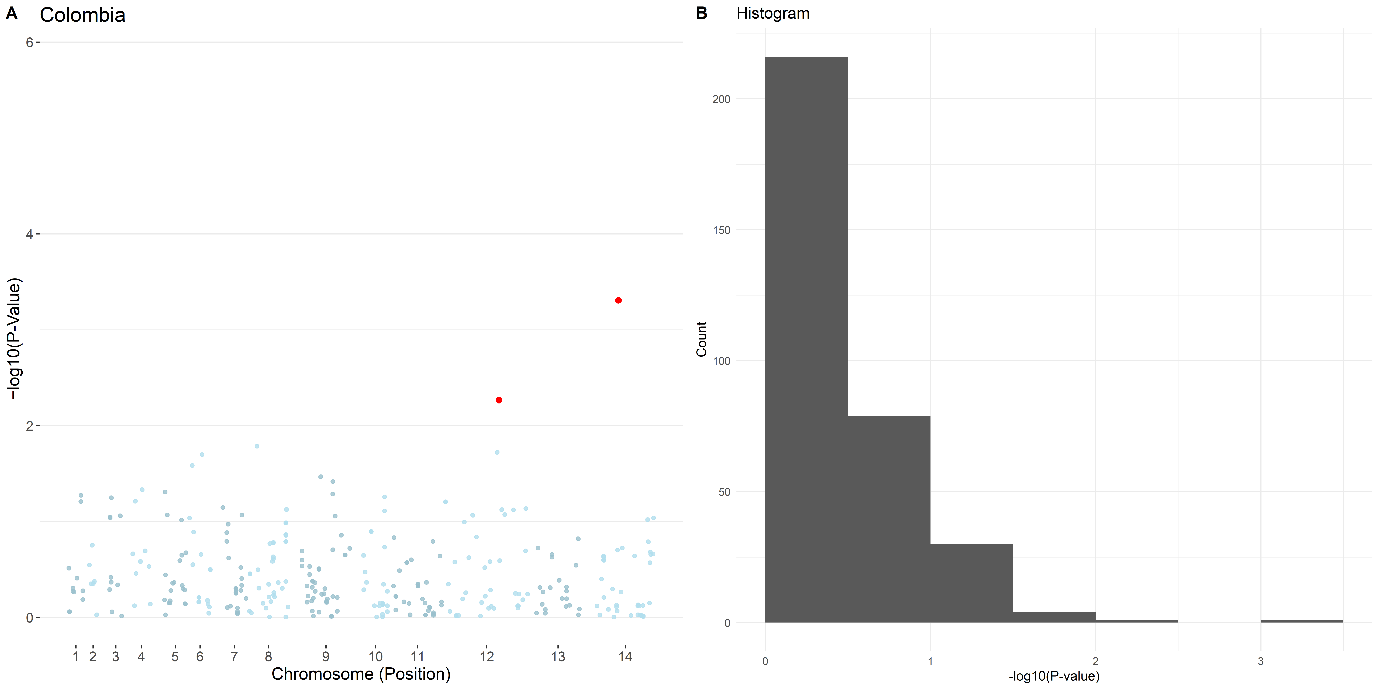
**

**Figure S18**

**Genome-wide XP-EHH analysis comparing *Plasmodium simium* and *P. vivax* from Panama.**(A) Manhattan plot showing XP-EHH scores across the 14 nuclear chromosomes of *P. vivax* PVP01. Each point represents a SNP positioned by its genomic location on the x-axis and the XP-EHH score on the y-axis. SNPs with significant signals of recent positive selection in *P. simium* relative to *P. vivax* from Panama are highlighted.
(B) Histogram depicting the distribution of p-values from the XP-EHH analysis across the genome, indicating the frequency of SNPs at varying significance levels.

**
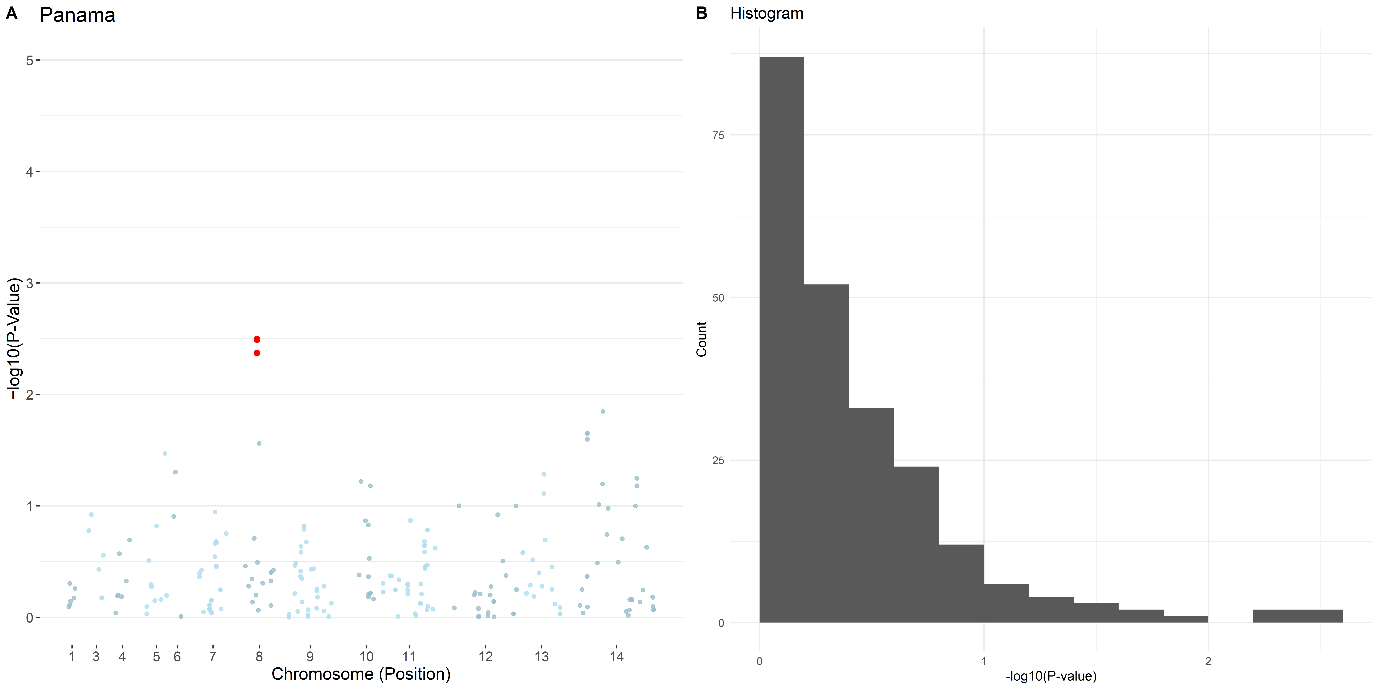
**

**Figure S19**

**Genome-wide XP-EHH analysis comparing *Plasmodium simium* and *P. vivax* from Peru.**(A) Manhattan plot showing XP-EHH scores across the 14 nuclear chromosomes of *P. vivax* PVP01. Each point represents a SNP positioned by its genomic location on the x-axis and the XP-EHH score on the y-axis. SNPs with significant signals of recent positive selection in *P. simium* relative to *P. vivax* from Peru are highlighted.
(B) Histogram depicting the distribution of p-values from the XP-EHH analysis across the genome, indicating the frequency of SNPs at varying significance levels.

**
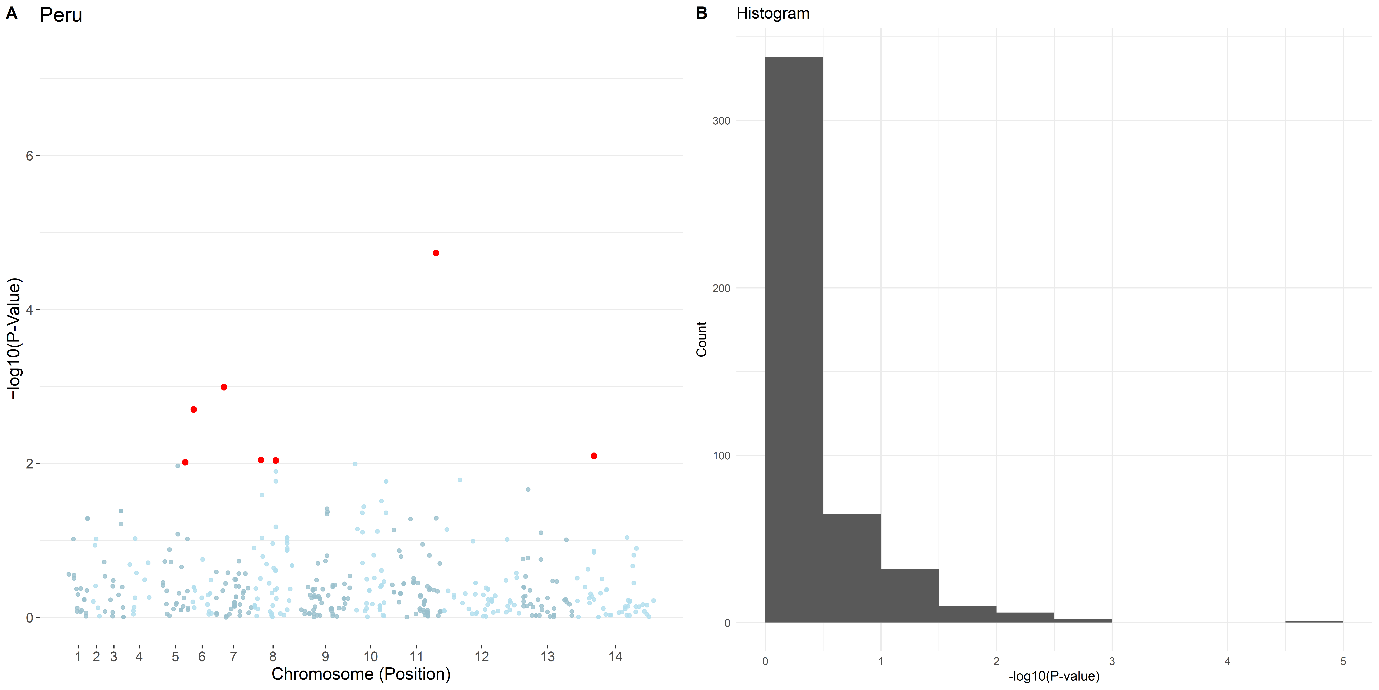
**
